# Influence of Igneous Basement on Deep Sediment Microbial Diversity on the Eastern Juan de Fuca Ridge Flank

**DOI:** 10.3389/fmicb.2017.01434

**Published:** 2017-08-02

**Authors:** Jessica M. Labonté, Mark A. Lever, Katrina J. Edwards, Beth N. Orcutt

**Affiliations:** ^1^Bigelow Laboratory for Ocean Sciences, East Boothbay ME, United States; ^2^Department of Marine Biology, Texas A&M University at Galveston, Galveston TX, United States; ^3^Center for Geomicrobiology, Aarhus University Aarhus, Denmark; ^4^Environmental Systems Science, ETH Zürich Zurich, Switzerland; ^5^Department of Biological Sciences, University of Southern California, Los Angeles CA, United States

**Keywords:** deep biosphere, IODP, basalt, sediment, Census of Deep Life

## Abstract

Microbial communities living in deeply buried sediment may be adapted to long-term energy limitation as they are removed from new detrital energy inputs for thousands to millions of years. However, sediment layers near the underlying oceanic crust may receive inputs from below that influence microbial community structure and/or activity. As part of the Census of Deep Life, we used 16S rRNA gene tag pyrosequencing on DNA extracted from a spectrum of deep sediment-basement interface samples from the subsurface of the Juan de Fuca Ridge flank (collected on IODP Expedition 327) to examine this possible basement influence on deep sediment communities. This area experiences rapid sedimentation, with an underlying basaltic crust that hosts a dynamic flux of hydrothermal fluids that diffuse into the sediment. Chloroflexi sequences dominated tag libraries in all sediment samples, with variation in the abundance of other bacterial groups (e.g., Actinobacteria, Aerophobetes, Atribacteria, Planctomycetes, and Nitrospirae). These variations occur in relation to the type of sediment (clays versus carbonate-rich) and the depth of sample origin, and show no clear connection to the distance from the discharge outcrop or to basement fluid microbial communities. Actinobacteria-related sequences dominated the basalt libraries, but these should be viewed cautiously due to possibilities for imprinting from contamination. Our results indicate that proximity to basement or areas of seawater recharge is not a primary driver of microbial community composition in basal sediment, even though fluids diffusing from basement into sediment may stimulate microbial activity.

## Introduction

Over the past decade, several studies have expanded our understanding of the composition and function of “deep biosphere” microbial communities in deep marine sediments in both organic-rich (D’Hondt et al., 2004; Inagaki et al., 2006; Biddle et al., 2006, 2008; Lever et al., 2010) and organic-poor environments (D’Hondt et al., 2009; Kallmeyer et al., 2012; Røy et al., 2012). Studies of the form and function of microbial communities within or expelled from the deep oceanic crust are rarer and geographically more limited (Cowen et al., 2003; Thorseth et al., 2003; Huber et al., 2006; Santelli et al., 2008; Orcutt et al., 2011a; Lin et al., 2012; Jungbluth et al., 2013; Lever et al., 2013; Lee et al., 2015), even though this habitat type is spatially extensive and occupies a large volume of the crust (Bach and Edwards, 2003; Orcutt et al., 2011b). Recent studies have challenged traditional microbiological concepts of what it means for cells to be alive, active, or dormant (Hoehler and Jørgensen, 2013). Metabolically active cells (Orsi et al., 2013) that can respond to nutrient addition through growth (Morono et al., 2011) have been confirmed in organic-rich sediment, while investigations in organic-poor environments have confirmed the persistence of microbial activity and cells under extreme energy limitation all the way through the sediment column to the underlying basement (D’Hondt et al., 2009, 2015; Røy et al., 2012). Another study confirmed the persistence of microbial groups from initial deposition through burial down to 2.5 km below seafloor millions of years later, and documented strong decreases in microbial abundance at 40–60°C (Inagaki et al., 2015), supporting the notion that increased biomolecule damage combined with severe energy limitation may cause the upper temperature limit of life in subsurface sediments to be lower than in energy-rich surface environments (Lever et al., 2015).

While most of these studies have focused on what happens to microbial life as it is buried beneath increasing layers of sediment, several recent studies have also highlighted the impact that the underlying basement can have on stimulating microbial activity from below. Ocean bottom water is entrained into ocean crust as a result of hydrothermal circulation, undergoing fluid–rock reactions during transit through basement that can change the chemistry of the fluid (Elderfield et al., 1999; Wheat and Mottl, 2004). These altered fluids can then diffuse upward into the overlying sediment, potentially providing a source of electron acceptors, nutrients, and organic carbon compounds to deeply buried sediment microbial communities. For example, in organic-poor sediment environments, oxygen and nitrate diffuse upward from minimally altered oxic basement fluids into sediment to stimulate microbial activity (Ziebis et al., 2012; Orcutt et al., 2013; D’Hondt et al., 2015; Wankel et al., 2015), although it is currently unclear what the influence is on deep sediment microbial community structure at these sites. Likewise, sulfate diffusing upward from highly altered anoxic basement fluids into overlying sediment may stimulate anaerobic microbial activity (Expedition 301 Scientists, 2005; Engelen et al., 2008; Lever et al., 2010). However, the influence of these basement sources on the structure of the microbial community is not currently resolved.

**FIGURE 1:**
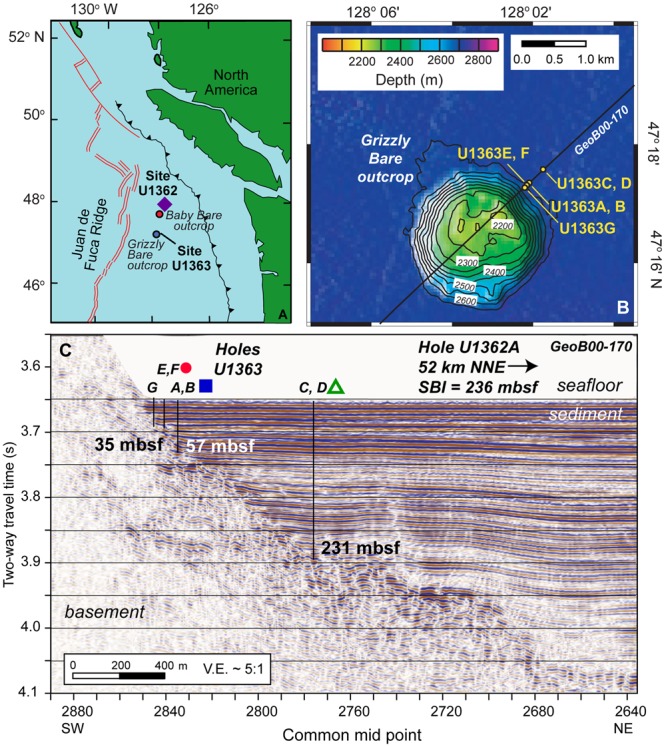
Maps of sampling locations discussed in this study. **(A)** IODP Expedition 327 coring locations Sites U1362 and U1363 on the eastern flank of the Juan de Fuca Ridge, shown in proximity to the Grizzly Bare and Baby Bare outcrops. **(B)** Plan view of Grizzly Bare outcrop coring locations located along seismic line GeoB00-170, with bathymetric relief as shown in legend. **(C)** Cross-section view of Grizzly Bare outcrop coring locations plotted on seismic profile, with depth of the sediment-basement interface (SBI) at study Holes indicated in meters below seafloor (mbsf). In A, purple diamond represents Hole U1362A; in C, red circle represents U1363F, blue square represents U1363B, and green triangle represents U1363D. Images modified from Fisher et al. (2011) and Wheat et al. (2013) with permission.

The Juan de Fuca Ridge flank is a well-studied ridge flank environment where bottom seawater recharges into young basement (3.5 Ma) at crustal outcrops and undergoes thermal and chemical alteration until its discharge as hydrothermal (∼64°C) fluids >50 km away (Wheat and Mottl, 2000; Wheat et al., 2013) (**Figure 1**). Sediment–basement chemical exchange in these locations is apparent from concentration profiles of sulfate, alkalinity, and calcium in the sediment (Supplementary Figure 1), as described elsewhere (Wheat et al., 2013). Increases in sulfate and calcium concentrations occur in bottom sediment toward the sediment-basement interface, indicating diffusion of sulfate and calcium from basement into overlying sediment. Bottom sediment also indicates the imprint of increasing chemical transformation of basement fluids during fluid–rock interaction: the increase in calcium concentration in bottom sediment along the flow path away from Grizzly Bare outcrop is an indication of the exchange reactions where magnesium is stripped from solution to replace calcium in crustal materials (Wheat et al., 2013; Lin et al., 2015).

In this study, we analyzed sediment microbial composition along a transect away from the edge of the Grizzly Bare seamount where seawater recharge occurs (**Figure 1** and **Table 1**), to examine whether microbial diversity in deep subsurface sediment is influenced by basement fluid–rock–sediment interactions. We also aimed to analyze microbial composition in deeply buried basalts from this environment, to see how rock-hosted microbial communities compare to recent studies on the communities found in the altered hydrothermal fluids and rock biofilms in borehole observatories (Cowen et al., 2003; Orcutt et al., 2011a; Baquiran et al., 2016; Jungbluth et al., 2016; Smith et al., 2016) and within microfissures of basalt (Lever et al., 2013). As is common for low biomass studies from deep marine environments (Inagaki et al., 2015; Lee et al., 2015), sequencing contamination presented a challenge to fully resolve basalt microbial communities, despite advances in DNA extraction methods (Lever et al., 2015); however, trends in sediment-basement connectivity were still addressable.

**Table 1:** Characteristics of the IODP Expedition 327 Juan de Fuca Ridge flank sampling locations discussed in this study.

ID	Location	Latitude (N)	Longitude (W)	Water depth (m)	SBI depth (mbsf)	SBI temperature (°C)
Hole U1362A	Second Rift	47.761047	127.761200	2661	236	64
Hole U1363B	Grizzly Bare Outcrop	47.289197	127.035100	2678	57	12
Hole U1363D	Grizzly Bare Outcrop	47.292873	128.029332	2678	231	33
Hole U1363F	Grizzly Bare Outcrop	47.288769	128.035623	2678	35	7

*mbsf, meters below seafloor; SBI, sediment-basement interface. Data summarized from previous reports (Expedition 327 Scientists, 2011b,c; Wheat et al., 2013).*

## Materials and Methods

### Sampling Sites

Samples were collected from the eastern flank of the Juan de Fuca Ridge (JdF; **Tables 1, 2** and **Figure 1**) during Integrated Ocean Drilling Program (IODP) Expedition 327 in Summer 2010 in a transect away from the Grizzly Bare outcrop (Expedition 327 Scientists, 2010, 2011c). Samples from Hole U1363F (**Figure 1B**) were collected in relatively shallow sediment (<33 m total sediment depth to the basement interface) on the flank of the outcrop. Samples from Holes U1362B and U1363D were collected farther from the edge in sediment roughly 55 and 231 m thick, respectively. These sediments ranged from hemipelagic mud (clays and silts) to sand-silt-clay turbidite sequences to carbonate-rich claystones (Expedition 327 Scientists, 2011c). By contrast, Hole U1362A (**Figure 1C**) was located ∼7 km north of the Baby Bare outcrop, a location of hydrothermal fluid discharge, and within a few 100 m of Sites 1026 and U1301 (Expedition 327 Scientists, 2011b). Samples from Hole U1362A represent basaltic oceanic crust that has undergone hydrothermal alteration based on the presence of abundant secondary clays, iron oxides, and localized pyrite deposits (Expedition 327 Scientists, 2011b). For this study, we focused on the deepest available sediment samples from Hole U1363B, D, and F, and two deeper basalt samples from Hole U1362A.

**Table 2:** Summary of IODP Expedition 327 sample depths, sediment sulfate concentration, sample descriptions, and potential for contamination (based on presence of fluorescent microspheres used during coring operations).

Core	Depth (mbsf)	SO_4_ (mM)	Description	μspheres^a^
U1363F-4H1	29.7	25.0	Clays	0
U1363F-4H2	31.2	25.6	Clays and Mn crusts	0
U1363F-4H3	31.9	26.2	Foram-rich and Mn crusts	0
U1363F-4HCC	32.3	25.2	Mn crusts, basalts, forams	1.3 × 10^4^
U1363B-8X2	50.2	26.6	Clays	0
U1363B-8X5	53.6	27.5	Foram-rich carbonate	0
U1363D-5X1	222.3	27.3	Clays	5.8 × 10^3^
U1363D-6XCC	231.2	n.a.	Altered massive basalt	n.m.
U1362A-16R2	456.7	n.a.	Altered massive basalt	0
U1362A-21R1A	491.4	n.a.	Basalt with chilled margin, exterior	7.9 × 10^1^
U1362A-21R1B	491.4	n.a.	Basalt with chilled margin, interior	0
U1362A-JdF control	n.a.	n.a.	Contamination check	n.m.

*n.a., not applicable; n.m., not measured; mbsf, meters below sea floor.*

*^a^Concentration of fluorescent microspheres per cm^3^ (wet sediment, interior of whole round core) or per g (rock), as presented elsewhere (Expedition 327 Scientists, 2011a,b,c).*

### Sample Collection and Handling

During IODP Expedition 327, hard rock samples for microbiological analysis were obtained by rotary core barrel (RCB) coring in Hole U1362A, and extended core barrel (XCB) coring at Site U1363 holes, as described in detail elsewhere (Expedition 327 Scientists, 2011a). Immediately following core delivery, rocks were exposed for subsampling in the core splitting room by shaking the recovered rocks into open split core liners. Rocks for microbiological sampling were identified immediately, photographed in place, and then collected using combusted aluminum foil for transport to the microbiology laboratory. There, rock pieces were transferred to a flame-sterilized stainless steel rock-processing box for subsampling. Following ethanol-rinsing and flame sterilization of the exterior, to remove potential contaminating organisms (Lever et al., 2006), rocks were broken into smaller pieces using flame-sterilized chisels and forceps. Portions of the interior and exterior were transferred into sterile vials containing distilled water for microsphere contamination analysis. The remaining rock pieces were transferred to sterile plastic Whirl-pak^®^ bags and frozen at -80°C until analysis.

Sediment samples for microbiological analysis on IODP Expedition 327 were collected by advanced piston coring (APC), or by XCB coring, as described in detail elsewhere (Expedition 327 Scientists, 2011a). Whole round core (WRC) of 10 cm length were cut on the catwalk and processed using sterilized tools (autoclaved spatulas and ethanol-cleaned end caps). Sterile plastic cut-end syringes were also inserted into the interior and exterior of freshly cut core ends to collect sediment for microsphere contamination analysis and cell density measurements, as described elsewhere (Expedition 327 Scientists, 2011c). The remainder of the WRC was transferred to sterile Whirl-pak^®^ bags, and frozen at -80°C. In the shore-based laboratory, frozen WRCs were subsampled with flame-sterilized tools, transferring the clean interior portions to sterile plastic vials for analysis.

### Contamination Testing

To examine potential contamination of hard rock and sediment cores collected during IODP Expedition 327, yellow–green, 0.2-μm-diameter fluorescent microspheres (Fluoresbrite Carboxylate Microspheres, Polysciences, Inc. 15700) were deployed in every core barrel according to standard IODP protocol (Smith et al., 2000), as described elsewhere (Expedition 327 Scientists, 2011a). Perfluorocarbon tracer contamination checks recommended elsewhere (Lever et al., 2006) were not available during this expedition due to use of the tracer pumping apparatus for other purposes. From the sediment cores, sterile cut-end 3 ml plastic syringes were inserted into the interior (center) and exterior (next to core liner) portions of freshly cut core sections, and sediment of a known volume was immediately fixed on the catwalk with 10 ml of cold 3.7% paraformaldehyde in 1× phosphate-buffered saline (PBS) buffer. From the rock samples, rock fragments of known weight were vigorously mixed with a few milliliters of distilled water to liberate microspheres from the rock surface. Samples were stored cold until analysis in a shore-based laboratory. Aliquots of the homogenized samples were filtered onto 25-mm-diameter 0.2-μm-mesh polycarbonate filters. Filters were examined under 100–400× total magnification with epifluorescence microscopy to quantify the number of fluorescent microspheres per samples.

To further evaluate the degree of potential contamination of the samples, a plastic bag that had been used to deliver the fluorescent microsphere solution in a core catcher during one of the basalt samplings, and which was then recovered in the core liner during basement coring operations, was preserved for DNA analysis (referred to as “JdF control”). The bag was removed from the core liner, transferred to a sterile Whirl-pak^®^ bags, and frozen at -80°C. This bag would have been exposed to contamination from human handling, seawater (drilling fluid matrix), drilling mud and drill pipe sources, as well as to the sample environment, and provides a background for evaluating potentially contaminating DNA.

As reported elsewhere (Expedition 327 Scientists, 2011c), the majority of samples showed no evidence of interior contamination, based on the absence of fluorescent microspheres (**Table 2**). Some interior samples from the sediment-basement interface sediment samples (i.e., U1363F-4HCC, U1363D-5X1) or from the exterior portion of a basement core (i.e., U1362A-21R1A) were positive for contamination from drilling fluids based on the presence of fluorescent microspheres.

### Cell Density Measurement

Following 6.5 years of storage at -20°C, cell densities in the non-core catcher sediment samples was determined on the same interior sediment samples used for fluorescent microsphere analysis described above, following published cell counting procedures (Kallmeyer et al., 2008). The formaldehyde-fixed sample slurry (1:6 sediment:buffer) was mixed with acid dissolution and detergent solutions to chemically release cells from the sediment matrix, and also gently sonicated to physically release cells from the matrix. Suspended cells were separated from sediment debris by density gradient centrifugation with Nycodenz. The cleaned cell pellet was concentrated on a 0.2-μm-mesh polycarbonate filter and stained with either acridine orange or propidium iodide DNA stains following methods described previously (Orcutt et al., 2011a), then visualized at 100x objective magnification on an Olympus BX60 epifluorescence microscope. Samples from the core catchers (i.e., U1363F-4HCC and U1363F-6HCC) were not preserved appropriately for direct cell counting.

### DNA Extraction

Environmental DNA from the JdF sediment (0.2–5 g), basalts (∼0.01–0.02 g), and the “JdF control” plastic bag (small square removed with sterile razor blade) was extracted using a combined mechanical and chemical lysis-based method (Lever et al., 2015). Negative extraction blanks were processed with each batch of samples. For the basalt samples, care was taken to only use material from interior fractures with evidence of alteration, which resulted in the rather small sample size. Using solutions containing the protein denaturants guanidium hydrochloride and tris(2-carboxyethyl)phosphine (TCEP), the surfactant Triton X-100, and dNTP, which minimized sorptive losses of DNA, cells were lysed by a combination of bead-beating, freeze-thawing, and incubation at 50°C, as per the published protocol. DNA extracts were then washed with chloroform-isoamylalcohol (24:1), precipitated with ethanol and linear polyacrylamide, and purified using the CleanAll DNA/RNA Clean-Up and Concentration kit (Norgen, Biotek, Corp., Canada). Attempts to extract DNA using the commercially available MO BIO PowerSoil^®^ DNA Isolation kit were unsuccessful. Quantification of DNA concentrations in the extracts was assessed using the Nanodrop PicoGreen quantification method according to manufacturer instructions.

### Quantitative PCR

Quantitative PCR (qPCR) was performed on a Light Cycler 480 Real-Time PCR System and LightCycler 480 SYBR Green I Master mix (both Roche Applied Science, Indianapolis, IN, United States). Two qPCR replicates were run for each sample. Bacterial 16S rRNA genes were amplified using the Bac8Fmod (5′-AGA GTT TGAT YMT GGC TCA G; Juretschko et al., 1998) and Bac338Rabc (5′-GCW GCC WCC CGT AGG WGT; Daims et al., 1999) primer combination. Archaea were amplified using the Arc915Fmod (5′-AAT TGG CGG GGG AGC AC; Cadillo-Quiroz et al., 2006) and Arc1059R (5′-GCC ATG CAC CWC CTC T; Yu et al., 2005) primer combination. Both primer pairs had been subjected to extensive tests, including *in silico*, melting curve and DNA sequence analyses on cloned qPCR products, to confirm specificity and good phylogenetic coverage of their target domains (Chen, 2014).

Quantitative PCR protocols consisted of 1 × 5 min denaturation at 95°C, 50× cycles of 30 s denaturation at 95°C, 30 s annealing at 55°C, 30 s elongation at 72°C, and 5 s fluorescence acquisition at 80°C, followed by melting curve analyses. qPCR standards were prepared from stock solutions of pGEM-T (Promega Corporation, Madison, WI, United States) plasmids, containing 16S rRNA genes of *Bacillus* sp. for Bacteria and *Methanosarcina* sp. for Archaea, that had been quantified using a NanoDrop 1000 spectrophotometer (NanoDrop Products, Wilmington, DE, United States).

The limit of detection, set by the extraction negative control and PCR negative control, ranged from 35 to 285 16S rRNA gene copies μL^-1^ of extract for Bacteria, and was <1 16S rRNA gene copy μL^-1^ of extract for Archaea. In any given run, samples in which 16S rRNA copy numbers of both sample PCR replicates exceeded those of both extraction and PCR negative control replicates by a factor of >2 were considered above detection. The mean of the highest negative control (could be either extraction or PCR negative, depending on the run), was then subtracted from the mean of sample replicates to calculate 16S rRNA gene copy numbers. The variability in background contamination was largely driven by the volumes of extraction reagents used per sample, which depended on the amount of sample material used during each DNA extraction.

### DNA Sequencing and Analysis

To examine the phylogenetic diversity of the microbial communities in these samples, the hypervariable V4–V6 region of the 16S rRNA gene in the environmental DNA extracts was targeted by 454 tag pyrosequencing. Tags were generated and sequenced at the Josephine Bay Paul Center at the Marine Biological Laboratory according to standard protocols using a 454 Life Sciences GS-FLX with Titanium chemistry (Sogin et al., 2006; Huse et al., 2007; Marteinsson et al., 2012) as part of the Deep Carbon Observatory’s Census of Deep Life. Sequencing included a Bacteria specific V4–V6 primer set (518F: 5′-CCAGCAGCYGCGGTAAN and 1064R: 5′-CGACRRCCATGCANCACCT), an Archaea specific V4–V6 primer set (517F: a mixture of 5′-GCCTAAAGCATCCGTAGC, 5′-GCCTAAARCGTYCGTAGC, 5′-GTCTAAAGGGTCYGTAGC, 5′-GCTTAAAGNGTYCGTAGC, and 5′-GTCTAAARCGYYCGTAGC and 1048R: 5′-CGRCRGCCATGYACCWC), and the Arch806F/Arch958R primer set (Delong, 1992), with 30 cycles of amplification with primer annealing at 60°C. The sequences were trimmed based on quality using the *mothur* software package as presented in the 454 Standard Operating Procedure (SOP) example analysis (Schloss et al., 2009, 2011). Following quality control, the sequences were trimmed to a minimum of 250 bp and a maximum of 350 bp to allow for a normalized sequence length for further processing.

To conservatively assess for the possibility of contamination of the samples from the drilling and/or handling processes, we followed an approach similar to recent reports (Inagaki et al., 2015; Lee et al., 2015). Sequences were clustered into operational taxonomic units (OTUs) at a >97% sequence similarity and then classified as recommended (Schloss et al., 2011) using the SILVA release version 119 full length and taxonomy reference alignment (Yilmaz et al., 2014) in *mothur*. The dataset was filtered using a conservative minimum OTU abundance cutoff threshold of 0.005% of total tags (five sequences), mitigating the generation of spurious OTUs (Bokulich et al., 2013); this filtering precludes calculating any richness estimates such as the Chao richness calculations that utilize number of singletons in calculations. The OTUs were then separated into four bins: (1) Control only – OTUs found only in the background contamination control (JdF control), (2) Samples only – OTUs found only in the basalt and sediment samples, (3) 10X in Sample – OTUs found in at least one sediment/basalt sample at >10× abundance as compared to the background sample, and (4) Control: Sample – OTUs found in at least one sediment/basalt sample at <10× abundance as compared to the background sample. All OTUs were also compared to the GenBank and RNA Database Project (RDP) databases to identify potential contaminants from DNA extraction and sequencing kits, as recommended elsewhere (Salter et al., 2014).

To examine the phylogeny of some OTUs, OTU sequences were compared with previously deposited sequences (SSU rRNA) using the RDP v10 Classifier (Cole et al., 2014) and National Center for Biotechnology Information (NCBI) BLASTnt nucleotide database (Altschul et al., 1997). The sequences were aligned with SINA (Pruesse et al., 2012). Phylogeny was performed using neighbor-joining implemented in Geneious (Biomatters, Auckland, New Zealand) with 1000 bootstrap replicated and the HKY model. Trees were constructed with FigTree^1^.

Raw sequence data are publicly available through the VAMPS Archive from the Josephine Bay Paul Center under project “DCO_ORC” as well as through the NCBI SRA at accession number SRP051698. Fasta formatted files for OTU bins are available in the Supplementary Material of this manuscript, as well as a table indicating OTU abundance (Supplementary Table 1).

## Results and Discussion

### Microbial Abundance Based on Cell Counting and qPCR

Microbial abundance in the sediment and basalt samples was analyzed in a variety of ways: direct cell counting using two different staining approaches, and quantitative assessment of 16S rRNA gene copy number in DNA extracts.

Interior sediment core samples were analyzed for cell density measurements following procedures developed for low biomass deep sediment samples (Kallmeyer et al., 2008). Cell density measurements were not performed on basalt samples or core catcher samples, as these samples were not preserved in an appropriate manner for this analysis. Two different cell-staining approaches were used – based on acridine orange (AODC) and prodium iodide (PI) DNA-staining agents – to assess the influence of cell staining technique on cell quantification. In the six sediment samples, cell densities ranged from 2–6 × 10^6^ cells cm^-3^ to 4–10 × 10^7^ cells cm^-3^ using the AODC and PI staining methods, respectively (**Table 3**). These values are within the ranges estimated previously for nearby sediment samples from IODP Hole U1301A using microscopic cell staining approaches (Engelen et al., 2008). The difference in cell densities estimated with the two staining approaches is unknown. As all cells were fixed with formaldehyde and rendered dead, difference in staining should not be due to exclusion of stains based on cell membrane integrity. Both stains suffer from non-specific binding to non-cell debris – despite attempts to separate and concentrate cells away from sediment particles using density centrifugation – which can make objective counting of small cell-sized particles challenging.

**Table 3:** Summary of cells and sequence groups in IODP Expedition 327 samples, including density of cells per cubic centimeter of sample as measured using acridine orange (AODC) and propidium iodide (PI) staining methods, the concentration of bacterial and archaeal 16S rRNA genes per gram sample, the number of sequences that passed initial quality control (QC seqs), the number of predicted operational taxonomic units at the 97% or greater sequence similarity threshold (# OTUs), and the number of OTUs in each sample after screening for potential contamination (# OTUs-control).

Core	Cells cm^-3^ AODC	Cells cm^-3^ PI	Bacteria 16S^b^ (copies/g)	Archaea 16S^c^ (copies/g)	QC seqs^d^	# OTUs^e^	# OTUs-control
U1363F-4H1^∗^	5 ± 3 × 10^6^	5 ± 2 × 10^7^	3.1 × 10^5^	2.3 × 10^4^	4,547	128	115
U1363F-4H2	4 ± 3 × 10^6^	5 ± 1 × 10^7^	2.4 × 10^5^	8.6 × 10^3^	15,582	257	239
U1363F-4H3^∗^	3 ± 3 × 10^6^	5 ± 3 × 10^7^	8.4 × 10^5^	2.7 × 10^4^	7,086	100	93
U1363F-4HCC	n.m.^a^	n.m.	1.4 × 10^7^	6.4 × 10^2^	15,479	10	3
U1363B-8X2	4 ± 3 × 10^6^	1 ± 0.4 × 10^8^	2.2 × 10^5^	2.9 × 10^3^	12,882	185	169
U1363B-8X5	6 ± 4 × 10^6^	4 ± 2 × 10^7^	4.9 × 10^3^	1.3 × 10^2^	10,634	89	65
U1363D-5X1	2 ± 2 × 10^6^	9 ± 4 × 10^7^	4.2 × 10^3^	3.1 × 10^3^	11,837	119	96
U1363D-6XCC	n.m.	n.m.	6.9 × 10^5^	3.0 × 10^3^	11,344	21	6
U1362A-16R2	n.m.	n.m.	1.1 × 10^6^	2.3 × 10^3^	9,081	24	10
U1362A-21R1A	n.m.	n.m.	1.1 × 10^6^	ND	9,057	24	11
U1362A-21R1B	n.m.	n.m.	1.4 × 10^6^	2.3 × 10^3^	8,232	21	3
U1362A-JdF control	n.m.	n.m.	5.8 × 10^6^	6.4 × 10^4^	3,732	164	116^f^

*^a^n.m., not measured.*

*^b^Concentration of bacterial 16S rRNA genes determined via QPCR with the Bac8Fmod-Bac338Rabc primer pair.*

*^c^Concentration of archaeal 16S rRNA genes determined via QPCR with the Arc915Fmod-Arc1059mod primer pair.*

*^d^Number of sequences longer than 250 bp after trimming based on quality scores.*

*^e^Number of OTUs represented by >5 tags in total.*

*^f^Number of OTUs found only in the JdF control sample.*

*^∗^Amplification with both Bacteria and Archaea primers.*

Following DNA extraction from 0.02 to 5 g sample, bulk DNA concentrations were quantifiable with the Nanodrop-3300 from only the four shallowest sediment samples (U1363F-4H1, U1363F-4H2, U1363F-4H3, and U1363B-8X2; **Table 3**). Despite using optimized methods for DNA extraction (Lever et al., 2015), DNA concentrations in the extracts from basalts and deep sediments were below the limit of detection of ≤10^3^ cells for a sample aliquot of 1 g. Despite the low DNA concentrations in the extracts, quantitative PCR analysis of bacterial and archaeal 16S rRNA genes abundance was robustly determined in comparison to template blanks and negative PCR extraction controls. Bacterial 16S rRNA genes were more abundant than archaeal 16S rRNA genes in all of the samples (**Table 3**). In fact, in none of the three basalt sample extracts were archaeal gene copy numbers above the operational detection limit of >2 times higher gene copies compared to extraction and PCR negative controls.

Sediment samples contained between 4.2 × 10^3^ and 8.4 × 10^5^ bacterial 16S rRNA gene copies per gram, with the exception of one sediment-basement interface sample (U1363F-4HCC) that had 10^7^ copies per gram. However, this one sample was recovered in the core catcher, visibly handled on the catwalk, and had abundant microspheres present; thus, this high gene copy estimate likely reflects external contamination during sampling or sample handling. The basalt samples had bacterial 16S rRNA gene copy densities of 10^6^ copies per gram of vein scraping; however, note that the samples focused on the alteration veins in the samples that likely have higher biomass densities than the bulk rock. Assuming an average RNA operon copy number per bacterial cell of 4 (Stoddard et al., 2015) and a sediment density of 2.5 g cm^-3^, the 16S rRNA gene copies per gram (**Table 3**) converts to a sediment cell density of 1 × 10^4^–2 × 10^6^ cells cm^-3^, which is within the low end of the range estimated for other deep sediments (Kallmeyer et al., 2012) and lower than previous direct cell counts in nearby sediment (Engelen et al., 2008). Archaeal rRNA gene copy numbers in sediments were one or more orders of magnitude lower (6.4 × 10^2^–8.4 × 10^5^) than bacterial rRNA gene copies and decreased toward the basaltic basement.

The low cell biomass predicted by qPCR for the sediments samples (**Table 3**) in combination with the lack of amplification of genes in the negative controls provides confidence that contamination of the DNA extracts with exogenous DNA during DNA extraction was unlikely. The approximately two order of magnitude difference in biomass estimates between the microscopic cell counts and qPCR-based approach (**Table 3**), which is a similar pattern to previous observations in nearby sediment (Engelen et al., 2008), may reflect possible overestimation of cell counts based on microscopy due to interference with other stained debris, as discussed above, or underestimation from the qPCR estimates due to inefficient DNA extraction or amplification inhibition. These differences in estimation method are important to consider when scaling such results to construct global inventories of deep biosphere biomass (Kallmeyer et al., 2012). Similarly, the estimates of biomass density in the basalt samples is a reflection of the density within the vein material, and not across the bulk basalt, so appropriate scaling of vein density would be required to convert these numbers to estimates of biomass within the basaltic crust for similar global estimates.

### DNA Sequence Analysis, OTU Determination, and Evaluation of Potential Contamination

All samples generated sequences with the Bacteria-specific V4–V6 region 16S rRNA gene primer set (**Table 3**), and two samples (U1363F-4H1 and U1363F-4H3) also generated sequence product with the Archaea specific V4–V6 primer set (marked with asterisk in **Table 3**). Amplification of the archaeal 16S rRNA gene with the Arch806F/Arch958R primer set was not successful (data not shown). Samples yielded between 3,732 and 15,479 quality V4–V6 tags following quality control (**Table 3**). A total of 1,394 OTUs with a >97% sequence similarity threshold were identified. After filtering for possibly spurious OTUs, 587 of these OTUs were comprised of >5 tags and were retained for further analysis (Supplementary Table 1).

To assess for possible contaminant sequences in the sample libraries – derived from either collection, handling, or sequence amplification sources – all of the 587 OTUs that passed quality control were divided into four sequence groups: (1) OTUs found only in the contamination JdF control (control only), (2) OTUs that were only present in samples (samples only), (3) OTUs that were at least 10 times more abundant in samples than in control (normalized to a common number of sequences per sample) (10X sample), and (4) OTUs that were less than 10 times more abundant in samples than in controls (control:sample). The third group (10X sample) represents cells that are relatively more abundant in the samples than in the control, or that preferentially over-amplified in the samples compared to the control. The fourth group (control:sample) represents cells that are found in both the samples and in the control. All sequences found in groups 3 and 4 could have been transferred from the subsurface environment to the control during collection or during laboratory handling.

**FIGURE 2:**
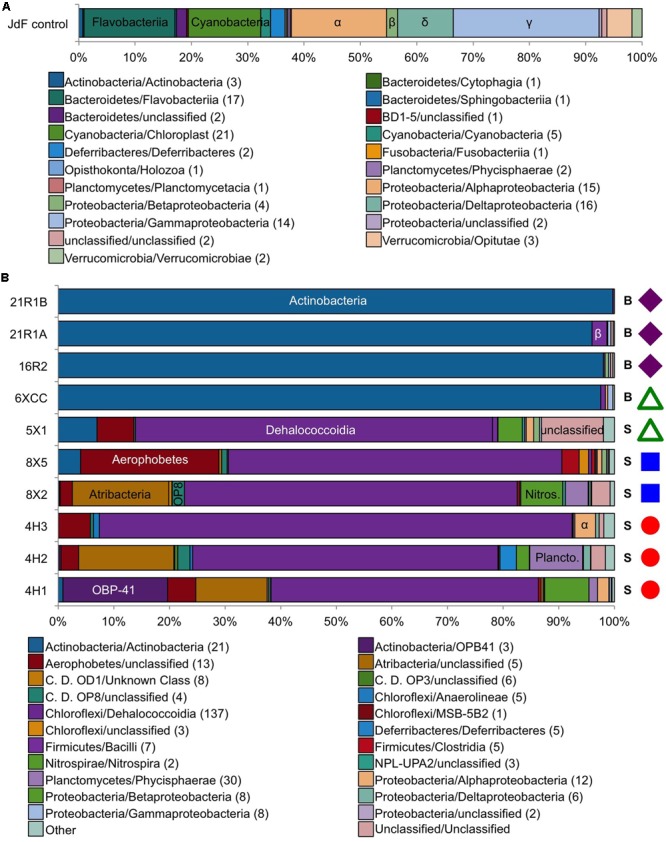
Taxonomic distribution at the class level of the bacterial tag sequences found in **(A)** the JdF control sample only (control only) and **(B)** in the sediment/basalt samples only (samples only). Colored symbols on right-hand side in B indicating sample location the same as in **Figure 1**. Number in parentheses in legend indicates how many OTUs are in each class. S, sediment; B, basalt.

**FIGURE 3:**
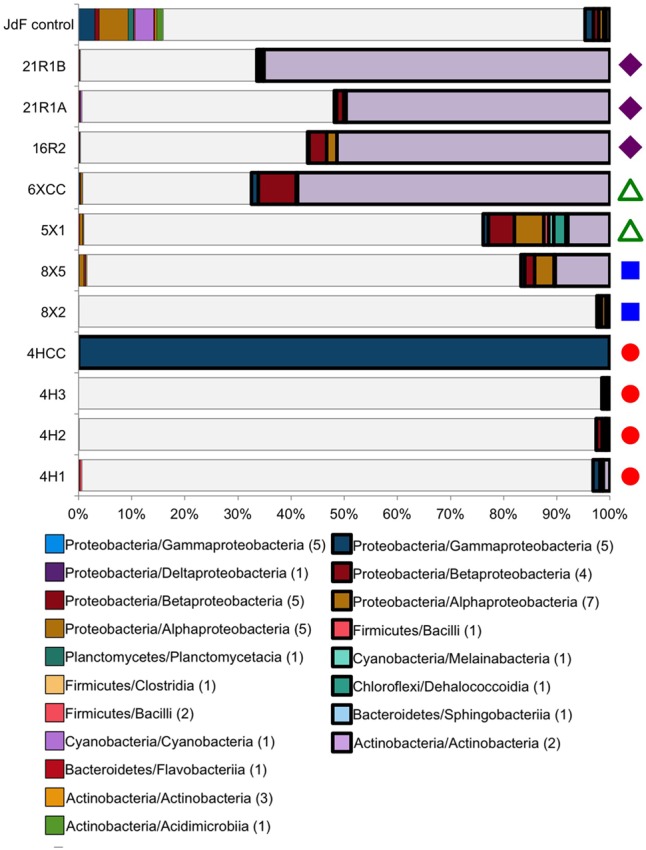
Taxonomic distribution at the class level of the Bacteria tag sequences found in both the JdF control sample (a plastic bag used to deliver the fluorescent microsphere solution in a core catcher during basalt coring) and the sediment and basalt samples. Taxonomic groups that are present in both the control and samples, and highly abundant in the sediment/basalt samples (i.e., 10X more sequences than in JdF control, 10X sample), are represented in bold on the right side. Taxonomic groups that are in relatively the same abundance in both the sediment/basalt samples and in the JdF control are on the left side (control:sample). The sequences that were unique to samples are represented in white, indicating the fraction of sequences that were kept for comparison analyses (samples only). Number in parentheses in legend indicates how many OTUs are in each class. Colored symbols on right-hand side in B indicating sample location the same as in **Figure 1**.

The JdF control sample generated 3,732 bacterial sequence reads after quality control filtering, which clustered into 164 OTUs at 97% sequence similarity level, with 116 OTUs unique to the control and considered as sequence group 1 (**Figure 2A** and **Table 3**). No archaeal sequences were recovered from the JdF control sample from either primer set, even though Archaeal 16S rRNA genes were detected via qPCR (**Table 3**). The majority of the unique-to-the-control bacterial OTUs grouped within the Gammaproteobacteria (25.6% of reads), Alphaproteobacteria (16.8%), Flavobacteriia (Bacteriodetes; 16.0%), and Cyanobacteria (12.8%) classes, with minor contributions from other classes. The majority of these OTUs are similar to bacteria commonly found in seawater, as demonstrated by the three most abundant OTUs belonging to SAR86 (Gammaproteobacteria; Dupont et al., 2012), SAR11 (Alphaproteobacteria; Rappé et al., 2002), and Flavobacteria NS4 group (Alonso et al., 2007). The dominant bacterial phyla in sequence group 1 were markedly different from the dominant phyla observed only in the sediment and basalt samples as sequence group 2 (**Figure 2**). The remaining 48 OTUs from the control sample that comprised about 20% of the sequences (**Figure 3**), and which were also observed in the samples as sequence groups 3 and 4, were predominantly phylogenetically related to known sequencing kit reagent contamination or to common seawater groups, as discussed below.

Between 2 and 100% of the bacterial sequences from each sediment and basement sample (**Figure 3**) had overlap with the JdF control sample [i.e., binning into sequence group 3 (10X sample) or 4 (control:sample); **Figure 3**], with higher overlap in samples with lower DNA extract concentrations (i.e., the deepest sediment samples and the basalts). Sequence group 3 was much more abundant in the sediment and basalt samples than sequence group 4, which made up <0.5% of sequences (**Figure 3**). The sequence group 4 OTUs (control:sample) were phylogenetically related to microbial groups that are commonly found in seawater, suggesting that these sequences could represent slight seawater contamination of the samples, or microbial groups that are common in seawater, sediment, and/or the basalt subsurface. Because of the possibility of these sequences potentially representing cross-contamination of seawater-derived groups during sample handling or sequencing, they were removed from further consideration of microbial community structure.

All of the sequence group 3 (10X sample) OTUs (shown in bold boxes in **Figure 3**) had high sequence similarity with common cultured isolates known as laboratory contaminants of DNA extraction kits and laboratory reagents (Salter et al., 2014) (Supplementary Table 1). For example, the abundant Actinobacteria OTUs from this sequence group in the basalt were closely related to *Arthrobacter* and *Rhodococcus* isolates from laboratory clean rooms and humans, respectively (Supplementary Figure 2). As another example, Firmicutes-related OTUs were observed in all of the samples, although in relatively low abundance (**Figure 3** and Supplementary Table 1). The majority of these OTUs grouped with known laboratory contaminants from human metagenomes (Bacillales, Clostridiales, Lactobacillales, and Selenomonadales) and other suspect contaminant groups (Ruminococcaceae, Halanaerobiales 64K2). The sequence library from sample U1363F-4HCC, which tested positive for contamination with the fluorescent microspheres (**Table 2**), was highly enriched (>99% of sequences) with Gammaproteobacteria Enterobacteriales OTUs also found in the background control (**Figure 3**). Thus, the sequence group 3 OTUs were categorized as probable sequencing contaminants and excluded from further analyses of microbial community structure; all of the sequences from the suspect U1363F-4HCC sample were not considered further. In summary, only sequence group 2 (samples only) sequences were considered for further microbial community structure analysis of the sediment and basalt samples.

### Sediment Microbial Community Structure

The sediment sample sequence libraries displayed little overlap with the background control sample that was collected during basalt coring operations (**Figures 2B, 3**). The majority of sequences grouped within the Chloroflexi phylum (60.3–86.7% of all QC reads; **Figure 2B** and Supplementary Table 1). The Chloroflexi-related OTUs grouped into seven different defined classes and two unclassified groups, with the majority of these grouping in the Dehalococcoidia class (**Figure 2B** and Supplementary Table 1). Phylogenetic analysis of all 136 Dehalococcoidia-related OTUs showed that all were highly similar to environmental sequences from other sediment environments and distinct from cultured isolates (Supplementary Figure 2). In an earlier clone library-based study from sediment squeeze cakes and resulting porewater from Hole U1363B deep sediment samples, Chloroflexi sequences were also detected, as well as in deeper re-sequencing of some of those samples (Jungbluth et al., 2013, 2016). Chloroflexi from the terrestrial subsurface are known to be involved in dehalogenation as a metabolic process, and may include sulfur cycling (Wasmund et al., 2016), but the function of Chloroflexi in organic-rich deep sediment are not verified (Kaster et al., 2014; Wasmund et al., 2014; Fullerton and Moyer, 2016).

Diverse Proteobacteria were observed in all sediment samples at relatively low abundance (<5% of QC tags; **Figure 2B** and Supplementary Table 1); however, the phylogenetic classification of some of these OTU sequences suggests that they should be viewed with caution. For example, Alphaproteobacteria-related sequences were the most abundant, grouping within the Rhizobiales and Rhodospirallales (**Figure 2B**). Rhizobiales are commonly found in soils, and the closest environmental relatives of the Rhodospirallales are from oxic/suboxic sediment from the South Pacific Gyre. Similarly, the Betaproteobacteria tags were related to Burkholderiales, which is known as a water filtration contaminant that can stymie environmental genomics surveys, although it is also found in sediment. The Gammaproteobacteria sequences were most closely related to Xanthomonadales, which is known to be enriched in drilling fluids (Masui et al., 2008). Only the Deltaproteobacteria sequences, grouped into five OTUs related to Desulfobacterales, were closely related to environmental sequences from anaerobic, sulfate-reducing sediment.

Other bacterial groups in relatively high abundance in some sediment samples included the Planctomycetes, Nitrospirae, and the recently described Aerophobetes and Atribacteria groups of uncultivated organisms (**Figure 2B** and Supplementary Figure 3). Planctomycetes had higher abundance in the clay-rich samples, and the majority of the sequences grouped mostly closely to Phycisphaerae clade MSBL9 observed in other deep sediment samples (Inagaki et al., 2006; Durbin and Teske, 2011), where they can be associated with fermentation at the sulfate-methane transition zone (Harrison et al., 2009). This same Planctomycetes group was also observed in an earlier clone library from Hole U1362B deep sediment (Jungbluth et al., 2013). Two Nitrospirales order OTUs were present in all clay samples, but the function of Nitrospirae in marine sediment is unknown. Nitrospirae are indicator taxa of altered crustal fluid in this environment (Jungbluth et al., 2016). While they were not detected in previous studies of sediment from the Juan de Fuca Ridge flank (Jungbluth et al., 2013, 2016), the sequences in our dataset are quite divergent from the sequences identified in the crustal fluid (78% similarity). Similarly, Atribacteria (candidate division JS-1) OTUs were in high abundance in the shallower clay samples from Hole U1363F and U1363B (∼17% of all QC tags), although not in the deeper clays from Hole U1363D. This group was also observed in earlier clone libraries from Hole U1363B deep sediment (Jungbluth et al., 2013) as well as recent re-sequencing of those DNA extracts (Jungbluth et al., 2016). Atribacteria are common in anaerobic, organic-rich sediment, where they are thought to be involved in organotrophic processes including fermentation (Dodsworth et al., 2013; Carr et al., 2015). All sediment samples had Aerophobetes sequences (previously known as BH180-139), with the highest concentration in the foram-rich carbonate sample U1363B-8X5 (24.8% of all QC sequences). This group was also detected in an earlier clone library of a deep sediment sample from Hole U1363B, as well as in shallower samples (Jungbluth et al., 2013, 2016). Very little is known about Aerophobetes except the suggestion that they may be strict anaerobes (Rinke et al., 2013), which could explain their higher abundance in deeper samples. Other Bacteria phyla were observed in the sediment samples, but never in high abundance (<5%, at most, of all Bacteria tags in a sample, Supplementary Table 1). These rare phyla included Acidobacteria, Bacteriodetes, Gemmatinomadetes, Spirochaetes, and candidate divisions BRC1, KB1 (Calescamantes), OD1, OP11, OP3, OP8 (Aminicenantes), WS3, and TA06. Although sediment samples shared many of the same bacterial groups, there was sample-specific clustering of the OTUs found in these phyla (Supplementary Figure 2).

**FIGURE 4:**
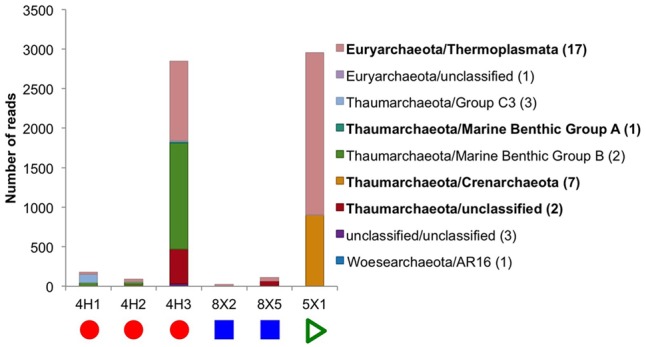
Taxonomic distribution at the class level of the Archaea tag sequences found in the sediment samples. No archaeal 16S rRNA genes could be sequenced from subseafloor basalt samples or the control. In bold are the classes that were found in higher abundance and are discussed in the main text. Number in parentheses in legend indicates how many OTUs are in each class. Due to the low number of reads in some samples, the graph is displayed as actual reads instead of being normalized to 100%. Colored symbols on at bottom indicating sample location the same as in **Figure 1**.

Six of the eight sediment samples yielded a few archaeal sequences from the sequencing runs with the Bacteria-specific V4–V6 primers, and two sediment samples (U1363F-4H1 and -4H3) generated thousands of QC sequences from the Archaea-specific primers (**Table 2**). Note that no archaeal sequences were recovered from the JdF control sample, as discussed above, so it is not possible to evaluate the potential for cross-contamination in this dataset. Both samples sequenced with the Archaeal-specific primer set yielded sequences related to Thermoplasmata and Thaumarchaeota, although the Thaumarchaeota groups were different (**Figure 4**). Previous clone libraries from deep sediment samples from Hole U1363B also detected Archaea, but from the Bathyarchaeota/Miscellaneous Crenarchaote Group (Jungbluth et al., 2013), which was not detected in the present study, while a more recent study of a “suite” of Site U1363 sediment samples also detected Marine Benthic Group E/Thermoplasmata (Jungbluth et al., 2016). The Archaea sequences picked up by the Bacteria-specific primer set also grouped within the Euryarchaeota and Thaumarchaeota (**Figure 4**). No sequences were recovered that were related to methanogens or anaerobic methanotrophs, which have been sequenced in prior studies of basalts from the Juan de Fuca subsurface using assays targeting the alpha subunit of the gene for methyl coenzyme M reductase (Lever et al., 2013).

**FIGURE 5:**
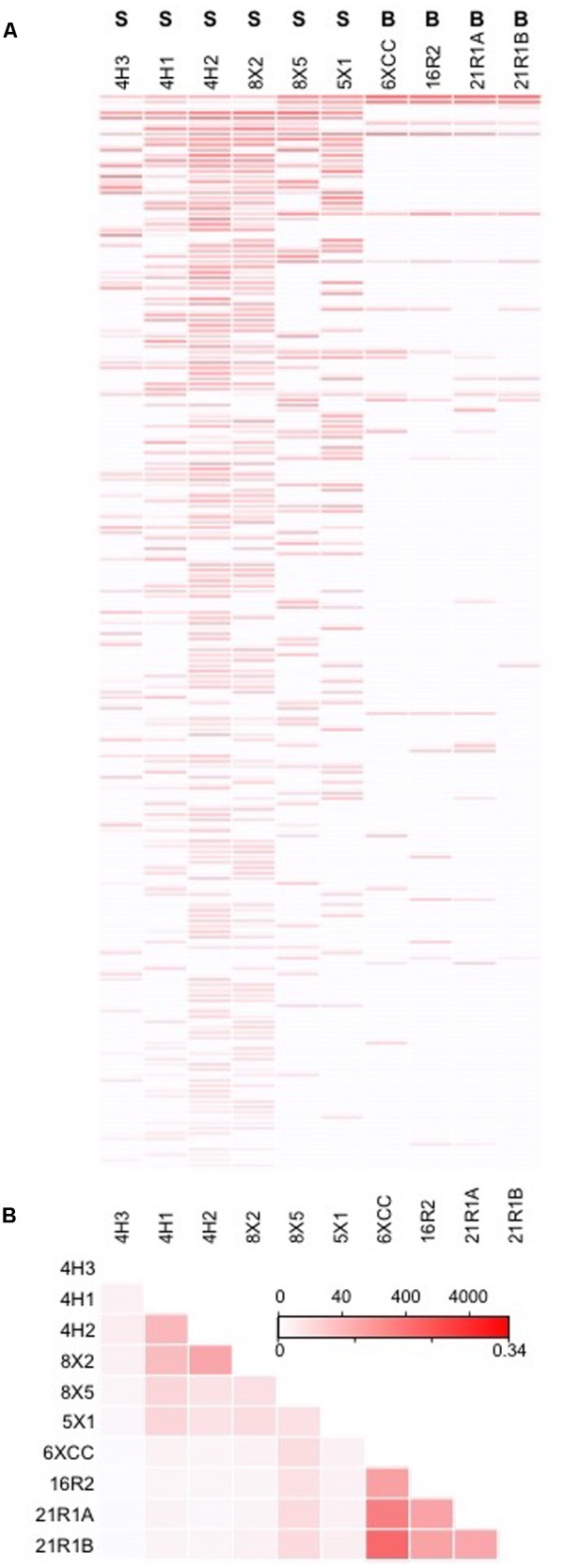
**(A)** Bacteria OTUs shared amongst more than one sample, ranked by abundance, and with color intensity reflecting abundance of OTU in the sample (top scale of legend in B indicating OTU abundance). **(B)** Heat map cross-comparison of sample community similarity using the Jaccard Index, with similarity levels scaled in bottom of legend.

### Basalt Microbial Community Structure

**FIGURE 6:**
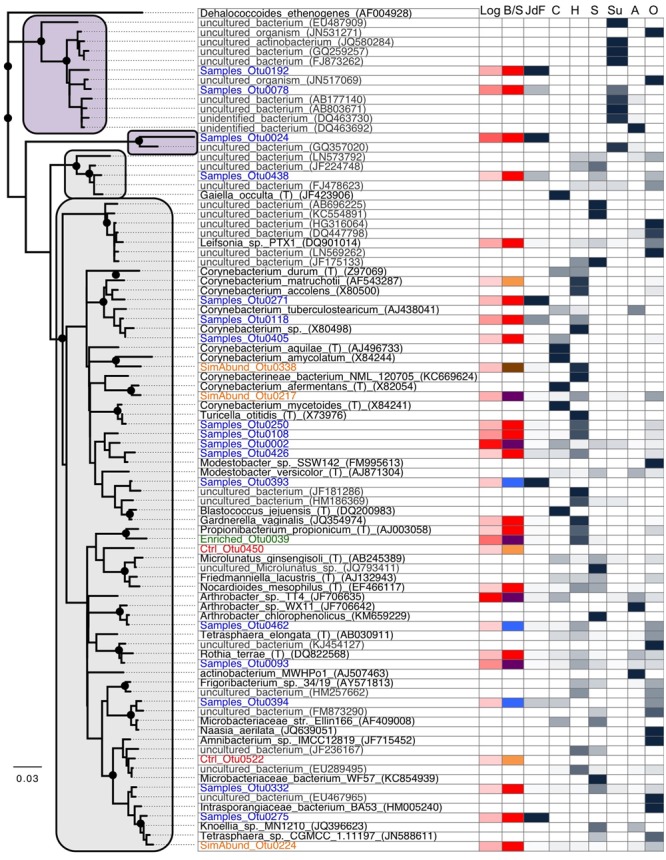
Phylogenetic analysis of the 16S rRNA gene from Actinobacteria OTUs from this study (Blue: samples only; Green: enriched in samples compared to control; Orange: similar abundance in samples and control; Red: control only), cultured microorganisms (black), and other closely related environmental sequences (gray). This tree shows the close phylogenetic relationship between the sequences from this study to common isolates as well as environmental sequences originating from human (mostly skin) and soil microbiome sequences, suggesting that the majority of the Actinobacteria-related sequences identified in our samples are laboratory contaminants. The tree was generated using neighbor-joining with 1000 bootstrap replicates, using the Jukes-Cantor model. Bootstrap replicates >90% are indicated by a black dot at the node. The first and second column represent the abundance of reads in this study (red; in log) and if the sequences were found in sediments only (blue), basalts only (blue), control only (orange), sediments and basalts (purple), or all three (brown). The remaining columns show the proportion of sequence found in each cluster that origin in our study (JdF), cultured microorganisms (C), human microbiome (H), soils (S), subsurface environments (Su), aquatic environments (A), or others (O). The majority of the sequences clustered into four supported groups where clades related to sediments are highlighted in purple and the others in gray.

The majority of sequences from the basalt samples grouped within sequence group 3 (i.e., they were also observed in the JdF control sample; **Figure 3**), and these were predominantly related to known isolates of Actinobacteria from clean room and human origin (**Figure 6** and Supplementary Table 1). Of the remaining sequences unique to the basalt samples (i.e., sequence group 2), the majority (<95%) of sequences were also similar to Actinobacteria (**Figure 2**). Less than 5% of the sequence group 2 basalt sequences grouped within other bacteria groups like the Firmicutes, Alphaproteobacteria, and Gammaproteobacteria (**Figure 2**). No archaeal sequences were recovered from the basalt samples.

Phylogenetic analysis of the Actinobacteria OTUs from this study compared to other studies provides a measure to assess the likelihood that an OTU represents a handling or sequencing contaminant or a true subsurface microbial group (**Figure 6**). Only three OTUs grouped only with sequences from other subsurface environments (i.e., purple box in **Figure 6**), and these three OTUs were only observed in sediment samples. The remaining Actinobacteria sequences in this study, including all of those from basalt samples, were most similar to either cultured microorganisms, or uncultured microorganisms found in various environments including human skin and soil/dust microbiomes (**Figure 6**). The most abundant OTU found in basalts (OTU0001, 56.1% of all reads from basalts; 2.8% of the reads from sediments) was found in the control sample as well (sequence group 3) and has >99% sequence similarity to the type strain isolate *Arthrobacter* sp. *TT4* (GenBank accession number JF706635; **Figure 6**), a strain isolated from a clean air space laboratory (Li et al., 2004). *Arthrobacter* is a common laboratory contaminant of reagents and DNA extraction kits (Salter et al., 2014). It is thus highly likely that this OTU represents a sequencing contaminant and should not be considered representative of the subsurface. In contrast, the second most abundant OTU in basalt samples (OTU0002; 37.5% of the basalt reads; 0.7% of the sediment reads) was not observed in the control sample but has >99% sequence similarity with the cosmopolitan strain *Rhodococcus erythropolis* (GenBank accession number X79289), which is known to cause infection in humans. Notably, this was the only OTU that was shared with another sample library from the Census of Deep Life – in particular, with sequences from a subseafloor basalt (data not shown; Jason Sylvan, personal communication). As this OTU was absent from our control sample and sequencing blanks analyzed in the other study, this may indicate that this *Rhodococcus*-related OTU has a subsurface origin, even though it has cosmopolitan distribution in disparate environments (**Figure 6**). All of the other Actinobacteria OTUs were far less abundant in the samples, and they also had phylogenetic similarity to isolates and uncultured species from various environments (**Figure 6**).

Resolving the subsurface and/or basaltic origin (or not) of the Actinobacteria from the basalt samples is challenging with the current dataset. Considering that our study only examined the 350 bp V4–V6 region of the 16S rRNA gene, it is possible that the OTUs with close V4–V6 region sequence similarity to sequences from dust and skin microbiomes may reveal variation when comparing larger sequence regions. Actinobacteria have been observed under cool and oxic conditions on seafloor and subseafloor basalts (Lysnes et al., 2004; Mason et al., 2009; Lee et al., 2015; Jørgensen and Zhao, 2016), in cool crustal fluids (Meyer et al., 2016), and in weathered volcanic glass (Cockell et al., 2009, 2013). Many of the Actinobacteria groups found in those cool and oxic studies, as well as in the current study (e.g., *Arthrobacter, Rhodococcus, Corynebacterium*) are cosmopolitan, and occur widely in terrestrial, marine, aeolian, animalian, oxic, and anoxic environments (**Figure 6**). For example, parsimony-based partial (non-overlapping) alignment of our V4–V6 tag sequences, the amplicon Actinobacteria sequences found in the recent analyses of the Mid-Atlantic Ridge flank basaltic subsurface (Jørgensen and Zhao, 2016; Meyer et al., 2016), and full length 16S rRNA gene data from environmental and cultured sequences reveals the cosmopolitan association of these groups, including the OTUs from this study that cannot be confirmed to be from the subsurface (data not shown). Actinobacteria have also been detected in the warm and anoxic crustal fluids from the Juan de Fuca (Jungbluth et al., 2012), but those specific lineages were not detected in the present study (data not shown).

Most known Actinobacteria have aerobic metabolisms, so finding them in the previous examinations of oxic environments is not surprising, whereas explaining their presence in the warm and anoxic JdF subsurface may be less straightforward. However, some Actinobacteria, including *Arthrobacter*, have been linked to anaerobic metabolisms including nitrate reduction and fermentation, both in the environment and in culture (Eschbach et al., 2003; Süß et al., 2006). Actinobacteria also have the ability to form spores, and could thus survive for long periods outside of suitable habitat without being metabolically active. Some Actinobacteria (Actinomycetes) form filaments, which could enable actinobacterial growth in rock environments in a manner previously suggested for Fungi (Ivarsson et al., 2016). Thus, Actinobacteria in anoxic deep biosphere environments could either be metabolically inactive survivors (e.g., remnants from aerobic surface sediment or seawater communities), or active in anaerobic metabolic processes. Further research is required to determine if subsurface Actinobacteria are metabolically active in subseafloor basalt, and the metabolic roles they play in this environment.

### Microbial Community Transitions in Basement-Influenced Sediment

Beyond determining the overall structure of the microbial communities in deep sediment and basement of the Juan de Fuca Ridge flank – which had not been fully addressed in previous studies of this system (Engelen et al., 2008; Lever et al., 2010, 2013; Jungbluth et al., 2013) – the aim of this study was to evaluate the influence of basement on the microbial ecology of deep sediment. Does deep sediment close to areas of seawater recharge into basement exhibit signs indicating that this influences the structure of the microbial communities? Or does the complex nature of fluid flow at outcrops, with recharge into basement occurring at the same time as discharge (Wheat et al., 2013; Winslow and Fisher, 2015), overprint this influence?

In this study, samples from Holes U1363F, U1363B, and U1363D progress in their distance away from the site of recharge (**Figure 1**), with samples U1363F-4H3, U1363B-8X5, and U1363D-5X1 being the sediment samples closest to the sediment-basement interface, thus comprising the set of samples that might display a trend. At a bulk level, these three samples are comprised of similar proportions of dominant Bacteria phyla – Chloroflexi are by the far most abundant sequence group, followed by Aerophobetes (**Figure 3**). However, at the “species”/OTU level of taxonomy, the deeper and farthest U1363D-5X1 sample appears to be comprised of a different constellation of OTUs as compared to the shallower and more proximal samples (**Figure 5**). While all of the sediment-basement interface samples share similar sulfate concentrations (26–27 mM, **Table 2** and Supplementary Figure 1), as well as similar concentrations of many other porewater constituents (Wheat et al., 2013), the shallower and more proximal samples have cooler temperatures (7–12°C) as compared to the distal and deeper location (33°C, Wheat et al., 2013), which may explain part of the variation in speciation. Notably, the deep sediments collected just a few 10s of meters away from the edge of net recharge outcrop do not indicate evidence of bottom seawater influence on microbial community structure following the conservative approach taken here (**Figure 2**), nor does the chemistry of sediment porewaters (Wheat et al., 2013). This is a conservative assessment, given that a few percent of sediment sequences (0.9–3.7%) fell into the potential seawater contaminant category, so comparison to the background sample may have artificially removed these sequences if they were present in both sediment samples and the background sample (**Figure 3**).

Because of our inability to confirm the origin of the sequences in the basalts, we cannot directly compare the sediment sequence libraries to the basalt sequence libraries to determine basement influence on sediment communities, and vice versa. However, recent deep sequencing work on highly altered crustal fluids from boreholes on the Juan de Fuca Ridge flank (Jungbluth et al., 2016) does provide an opportunity to assess the degree of basement fluid influence on basal sediment community structure. Although the fluid study was conducted on fluids in the vicinity of Hole U1362A, and not directly underneath the Site U1363 holes 52 km away, the porewater chemistry at Site U1363 is indicative of warm and anoxic fluids in basement similar to the Hole U1362A region. When comparing the sediment sample sequences in the present study to the sequences identified in the crustal fluids, the bacterial communities of the sediments are quite divergent from the indicator taxa identified in the crustal fluids, indicating a segregation between the microbial communities found in the sediments and in the fluids. For example, there was only 88–90% sequence similarity and <80% similarity to the indicator taxa Aminicenantes (OP8) and Calescamantes (BK1), respectively (data not shown).

The microbial communities living in deeply buried sediments have been cut off from new detrital energy inputs for thousands to millions of years and need to adapt to constant energy and nutrient limitation. The Juan de Fuca Ridge is influenced by hydrothermal activity and fast sedimentation rates, which influences energy availability (LaRowe and Amend, 2014). The Chloroflexi phylum dominated the sediment samples, suggesting that these microbes are well-adapted to organic rich anoxic sediments. However, there were differences amongst the less dominant groups (**Figures 2B, 5**), and these differences in the microbial communities could be explained by the physical and chemical characteristics of the sediments. Nitrospirae was more prevalent in clays, indicating that the type of material is an important part of microbial adaptation. Actinobacteria OBP1 was more prevalent only in one sample, U1363F-4H1, which is the shallowest sample and with the “freshest” organic matter. Atribacteria and Planktomycetes were present only in the clay samples that were closer to the recharge area, while Aerophobetes were more abundant in the clay samples farther from the recharge area. The sediments display a gradient of temperature, which could be the main factor for the changes in microbial communities. Finally, there are still many unknown groups of bacteria that inhabit these sediments that remain uncharacterized, with a higher proportion of unclassified tags in the deepest samples.

### Future Improvements for DNA-Based Assessment of Deep Sediment and Crust

Our DNA sequence data suggest DNA contamination in at least one of the sediment samples and all the basalt samples analyzed in this study. The sediment-basement interface sample of Hole U1363F (i.e., 4HCC), was contaminated during sample drilling and handling, as evidenced by the high concentration of fluorescently labeled microsphere at sampling, and the overwhelming dominance of Enterobacterales-related sequences (**Table 1** and **Figure 3**). The basalt samples and low biomass sediment samples (U1363B-8X5 and U1363D-5X1) were likely contaminated during sample handling, DNA extraction or sequencing due to their very low biomass, as demonstrated by the enrichment of common laboratory reagent and airborne contaminants. These observations confirm that even with improved methods for core handling and sample processing (Expedition 327 Scientists, 2011a), DNA contamination of deep biosphere samples remains a challenge (Santelli et al., 2010; Lever, 2013; Inagaki et al., 2015).

Based on our results, the use of fluorescent microsphere proved to be a useful – though not quantitative (House et al., 2003) – tool to detect drilling fluid contamination, but more efforts need to be made to properly address contamination in low biomass samples. DNA contamination was also an issue in recent sampling of subsurface basalts from North Pond (Jørgensen and Zhao, 2016; Zhang et al., 2016) and from other low biomass environments where over 99% of raw sequences were suspect (Inagaki et al., 2015). For future expeditions, we suggest taking water column and drilling fluid samples as contamination controls and sequencing both separately, to enable more reliable distinctions between DNA sequences in drilling fluids that were introduced from the subsurface environment from ones that were introduced from surface seawater or during core handling. We encourage the systematic sequencing of a blank DNA extraction control to correctly remove contaminants that are present in the DNA extraction and sequencing reagents, as was done during IODP Expedition 337 (Inagaki et al., 2015). For extremely low biomass basalt samples, it will be important to collect larger rock samples for DNA extraction, as was done in several other studies, where larger samples were available for microbiological analyses (Lever et al., 2013; Jørgensen and Zhao, 2016; Zhang et al., 2016). This will increase the amount of sample material from rock interiors, which tend to be minimally contaminated (Lever et al., 2006). Increasing the amount of suitable sampling material will enable lower detection sensitivity of *in situ* microbial communities, lower the risk of significant contamination by laboratory handling, and maximize the sequencing of DNA coming from the sample. Finally, we caution all users of publically available sequence datasets, such as those from the Census of Deep Life, to critically evaluate for the possibility of sequence contamination in the samples.

Another strategy that will improve the reliability of nucleic acid data and help identify DNA sequences from indigenous microorganisms is to combine multiple interdisciplinary approaches. In the past, DNA sequence data have been combined with geochemical (e.g., electron donor and acceptor availability), isotopic (providing clues to ongoing microbial metabolic processes), and mineralogical data (e.g., redox/oxidation state, olivine content), as well as laboratory enrichments to demonstrate microbial carbon and sulfur cycling in subseafloor basalt (Lever et al., 2013). Microbial colonization experiments involving different rock types, and combining molecular biological and microbiological analyses with monitoring of surrounding fluid chemistry and rock alteration processes in CORKS have provided new insights into microbial colonization patterns and how rock biofilms form (Orcutt et al., 2011a; Smith et al., 2011, 2016; Baquiran et al., 2016). Future studies on unaltered rock samples and after cultivation experiments may include single-cell genome, metagenome, and metatranscriptome sequencing, and compare potential metabolisms inferred from genomic information to data on potential *in situ* microbial activities obtained with other methodologies to distinguish *in situ* microorganisms from microorganisms that were likely introduced during sampling, sample handling, or any other downstream laboratory-based research activities.

## Conclusion

Our study provides a glimpse of microbial community dynamics and interactions across sediment-basement interfaces in the marine deep subsurface. Our results demonstrate that proximity of basal sediment to a site of seawater recharge into basement bears little correlation with sediment microbial community structure, which is more strongly influenced by depth below the seafloor and sediment type. Moreover, there appears to be little overlap in microbial community structure between basal sediments and basement fluids. The composition of basal sediment microbial communities is characteristic of anoxic, relatively organic rich subsurface sediment environments elsewhere, with dominance by subseafloor Chloroflexi, Aerophobetes, Atribacteria, and Nitrospirae. By contrast, the full phylogenetic composition of microbial biofilms within upper basement basalts from the Juan de Fuca subsurface remains elusive, unfortunately, due to typical contamination-related challenges of research on low biomass samples and due to strong phylogenetic overlaps not only with other basalt habitats but also with a wide range of other environments. Future studies involving deep sequencing of drilling fluids and all negative controls, using larger rock samples with a higher biomass of indigenous microorganisms inside, and complementing nucleic acid sequencing with other methodologies that provide insights into microbial energy sources and activities, will contribute to a more clear and accurate picture of the indigenous basalt microbiota.

## Author Contributions

BO and KE designed the project, BO collected the samples, ML performed laboratory analyses, JL and ML performed data analysis, and JL and BO wrote the paper with input from coauthors.

## Conflict of Interest Statement

The authors declare that the research was conducted in the absence of any commercial or financial relationships that could be construed as a potential conflict of interest.

## Footnotes

1 http://tree.bio.ed.ac.uk/software/figtree

## References

[B1] Alonso C. Warnecke F. Amann R. Pernthaler J. (2007). High local and global diversity of *Flavobacteria* in marine plankton. *Environ. Microbiol.* 9 1253–1266. 10.1111/j.1462-2920.2007.01244.x 17472638

[B2] Altschul S. F. Madden T. L. Schaffer A. A. Zhang J. H. Zhang Z. Miller W. (1997). Gapped BLAST and PSI-BLAST: a new generation of protein database search programs. *Nucleic Acids Res.* 25 3389–3402. 10.1093/nar/25.17.3389 9254694 PMC146917

[B3] Bach W. Edwards K. J. (2003). Iron and sulfide oxidation within the basaltic ocean crust: implications for chemolithoautotrophic microbial biomass production. *Geochim. Cosmochim. Acta* 67 3871–3887. 10.1016/S0016-7037(03)00304-1

[B4] Baquiran J.-P. Ramírez G. A. Haddad A. G. Toner B. M. Hulme S. Wheat C. G. (2016). Temperature and redox effect on mineral colonization in Juan de Fuca Ridge Flank subsurface crustal fluids. *Front. Microbiol.* 7:396 10.3389/fmicb.2016.00396 PMC4815438 27064928

[B5] Biddle J. F. Lipp J. S. Lever M. A. Lloyd K. G. Sørensen K. B. Anderson R. (2006). Heterotrophic Archaea dominate sedimentary subsurface ecosystems off Peru. *Proc. Natl. Acad. Sci. U.S.A.* 103 3846–3851. 10.1073/pnas.0600035103 16505362 PMC1533785

[B6] Biddle J. F. Fitz-Gibbon S. Schuster S. C. Brenchley J. E. House C. H. (2008). Metagenomic signatures of the Peru Margin subseafloor biosphere show a genetically distinct environment. *Proc. Natl. Acad. Sci. U.S.A.* 105 10583–10588. 10.1073/pnas.0709942105 18650394 PMC2492506

[B7] Bokulich N. A. Subramanian S. Faith J. J. Gevers D. Gordon J. I. Knight R. (2013). Quality-filtering vastly improves diversity estimated from Illumina amplicon sequencing. *Nat. Methods* 10 57–59. 10.1038/nmeth.2276 23202435 PMC3531572

[B8] Cadillo-Quiroz H. Bräuer S. Yashiro E. Sun C. Yavitt J. Zinder S. H. (2006). Vertical profiles of methanogenesis and methanogens in two contrasting acidic peatlands in central New York State, USA. *Environ. Microbiol.* 8 1428–1440. 10.1111/j.1462-2920.2006.01036.x 16872405

[B9] Carr S. A. Orcutt B. N. Mandernack K. W. Spear J. R. (2015). Abundant Atribacteria in deep marine sediment from the Adélie Basin, Antarctica. *Front. Microbiol.* 6:872 10.3389/fmicb.2015.00872 PMC4549626 26379647

[B10] Chen X. (2014). *Controls on Microbial Community Zonation in Coastal Marine Sediment (Aarhus Bay)*. Ph.D. thesis, Aarhus University Aarhus.

[B11] Cockell C. S. Kelly L. C. Marteinsson V. T. (2013). *Actinobacteria* - an ancient phylum active in volcanic rock weathering. *Geomicrobiol. J.* 30 706–720. 10.1080/01490451.2012.758196

[B12] Cockell C. S. Olsson K. Knowles F. Kelly L. C. Herrera A. Thorsteinsson T. (2009). Bacteria in weathered basaltic glass, Iceland. *Geomicrobiol. J.* 26 491–507. 10.1080/01490450903061101

[B13] Cole J. R. Wang Q. Fish J. A. Chai B. McGarrell D. M. Sun Y. (2014). Ribosomal Database Project: data and tools for high throughput rRNA analysis. *Nucleic Acids Res.* 42 D633–D642. 10.1093/nar/gkt1244 24288368 PMC3965039

[B14] Cowen J. Giovannoni S. J. Kenig F. Johnson H. P. Butterfield D. A. Rappé M. S. (2003). Fluids from aging ocean crust that support microbial life. *Science* 299 120–123. 10.1126/science.1075653 12511653

[B15] Daims H. Bruhl A. Amann R. Schleifer K.-H. Wagner M. (1999). The domain-specific probe EUB338 is insufficient for the detection of all Bacteria: development and evaluation of a more comprehensive probe set. *Syst. Appl. Microbiol.* 22 434–444. 10.1016/S0723-2020(99)80053-8 10553296

[B16] Delong E. F. (1992). Archaea in coastal marine sediments. *Proc. Natl. Acad. Sci. U.S.A.* 89 5685–5689. 10.1073/pnas.89.12.5685 1608980 PMC49357

[B17] D’Hondt S. Inagaki F. Zarikian C. A. Abrams L. J. Dubois N. Engelhardt T. (2015). Presence of oxygen and aerobic communities from sea floor to basement in deep-sea sediments. *Nat. Geosci.* 8 299–304. 10.1038/ngeo2387

[B18] D’Hondt S. Jørgensen B. B. Miller D. J. Batzke A. Blake R. Cragg B. A. (2004). Distributions of microbial activities in deep subseafloor sediments. *Science* 306 2216–2221. 10.1126/science.1101155 15618510

[B19] D’Hondt S. Spivack A. Pockalny R. Ferdelman T. Fischer J. Kallmeyer J. (2009). Subseafloor sedimentary life in the South Pacific Gyre. *Proc. Natl. Acad. Sci. U.S.A.* 106 11651–11656. 10.1073/pnas.0811793106 19561304 PMC2702254

[B20] Dodsworth J. A. Blainey P. C. Murugapiran S. K. Swingley W. D. Ross C. A. Tringe S. G. (2013). Single-cell and metagenomic analyses indicate a fermentative and saccharolytic lifestyle for members of the OP9 lineage. *Nat. Commun.* 4:1854 10.1038/ncomms2884 PMC3878185 23673639

[B21] Dupont C. L. Rusch D. B. Yooseph S. Lombardo M. J. Richter R. A. Valas R. (2012). Genomic insights to SAR86, an abundant and uncultivated marine bacterial lineage. *ISME J.* 6 1186–1199. 10.1038/ismek.2011.189 22170421 PMC3358033

[B22] Durbin A. M. Teske A. (2011). Microbial diversity and stratification of South Pacific abyssal marine sediments. *Environ. Microbiol.* 13 3219–3234. 10.1111/j.1462-2920.2011.02544.x 21895908

[B23] Elderfield H. Wheat C. G. Mottl M. J. Monnin C. Spiro B. (1999). Fluid and geochemical transport through oceanic crust: a transect across the eastern flank of the Juan de Fuca Ridge. *Earth Planet. Sci. Lett.* 172 151–165. 10.1016/S0012-821X(99)00191-0

[B24] Engelen B. Ziegelmueller K. Wolf L. Kopke B. Gittel A. Cypionka H. (2008). Fluids from the oceanic crust support microbial activities within the deep biosphere. *Geomicrobiol. J.* 25 56–66. 10.1080/01490450701829006

[B25] Eschbach M. Möbitz H. Rompf A. Jahn D. (2003). Members of the genus *Arthrobacter* grow anaerobically using nitrate ammonification and fermentative processes: anaerobic adaptation of aerobic bacteria abundant in soils. *FEMS Microbiol. Lett.* 223 227–230. 10.1016/S0378-1097(03)00383-5 12829291

[B26] Expedition 301 Scientists (2005). “Site U1301,” in *Proceedings of the IODP* Vol. 301 eds Fisher A. T. Urabe T. Klaus A. Expedition 301 Scientists (College Station, TX: Integrated Ocean Drilling Program Management International, Inc.).

[B27] Expedition 327 Scientists (2010). Juan de Fuca Ridge-flank hydrogeology: the hydrogeologic architecture of basaltic oceanic crust: compartmentalization, anisotropy, microbiology, and crustal-scale properties on the eastern flank of the Juan de Fuca Ridge, eastern Pacific Ocean. *IODP Prel. Rept.* 327 1–73.

[B28] Expedition 327 Scientists (2011a). “Methods,” in *Proceedings of the IODP* Vol. 327 eds Fisher A. T. Tsuji T. Petronotis K. Expedition 327 Scientists (Tokyo: Integrated Ocean Drilling Program Management International, Inc.).

[B29] Expedition 327 Scientists (2011b). “Site U1362,” in *Proceedings of IODP* Vol. 327 eds Fisher A. T. Tsuji T. Petronotis K. Expedition 327 Scientists (Tokyo: Integrated Ocean Drilling Program Management International, Inc.).

[B30] Expedition 327 Scientists (2011c). “Site U1363,” in *Proceedings of IODP* Vol. 327 eds Fisher A. T. Tsuji T. Petronotis K. Expedition 327 Scientists (Tokyo: Integrated Ocean Drilling Program Management International, Inc.).

[B31] Fisher A. T. Wheat C. G. Becker K. Cowen J. Orcutt B. Hulme S. (2011). “Design, deployment, and status of borehole observatory systems used for single-hole and cross-hole experiments, IODP Expedition 327, eastern flank of Juan de Fuca Ridge,” in *Proceedings of the IODP 327* eds Fisher A. T. Tsuji T. Petronotis K. Expedition 327 Scientists (Tokyo: Integrated Ocean Drilling Program Management International, Inc.).

[B32] Fullerton H. Moyer C. (2016). Comparative single-cell genomics of *Chloroflexi* from the Okinawa Trough deep subsurface biosphere. *Appl. Environ. Microbiol.* 82 3000–3008. 10.1128/AEM.00624-16 26969693 PMC4959059

[B33] Harrison B. K. Zhang H. Berelson W. Orphan V. J. (2009). Variations in archaeal and bacterial diversity associated with the sulfate-methane transition zone in continental margin sediments (Santa Barbara Basin, California). *Appl. Environ. Microbiol.* 75 1487–1499. 10.1128/AEM.01812-08 19139232 PMC2655439

[B34] Hoehler T. M. Jørgensen B. B. (2013). Microbial life under extreme energy limitation. *Nat. Rev. Microbiol.* 11 83–94. 10.1038/nrmicro2939 23321532

[B35] House C. H. Cragg B. A. Teske A. The Leg 201 Scientific Party. (2003). “Drilling contamination tests during ODP Leg 201 using chemical and parti1culate tracers,” in *Proceedings of the Ocean Drilling Program, Initial Reports* Vol. 201 eds D’Hondt S. Jørgensen B. B. Miller D. J. (College Station, TX: Ocean Drilling Program).

[B36] Huber J. A. Johnson H. P. Butterfield D. A. Baross J. A. (2006). Microbial life in ridge flank crustal fluids. *Environ. Microbiol.* 8 88–99. 10.1111/j.1462-2920.2005.00872.x 16343325

[B37] Huse S. M. Morrison H. G. Sogin M. L. Welch D. M. (2007). Accuracy and quality of massively parallel DNA pyrosequencing. *Genome Biol.* 8:R143 10.1186/gb-2007-8-7-r143 PMC2323236 17659080

[B38] Inagaki F. Hinrichs K. U. Kubo Y. Bowles M. W. Heuer V. B. Hong W. L. (2015). Exploring deep microbial life in coal-bearing sediment down to ∼2.5 km below the ocean floor. *Science* 349 420–424. 10.1126/science.aaa6882 26206933

[B39] Inagaki F. Nunoura T. Nakagawa T. Teske A. Lever M. Lauer A. (2006). Biogeographical distribution and diversity of microbes in methane hydrate-bearing deep marine sediments on the Pacific Ocean Margin. *Proc. Natl. Acad. Sci. U.S.A.* 103 2815–2820. 10.1073/pnas.0511033103 16477011 PMC1413818

[B40] Ivarsson M. Schnürer A. Bengston S. Neubeck A. (2016). Anaerobic fungi: a potential source of biological H_2_ in the oceanic crust. *Front. Microbiol.* 7:674 10.3389/fmicb.2016.00674 PMC4922220 27433154

[B41] Jørgensen S. L. Zhao R. (2016). Microbial inventory of deeply buried oceanic crust from a young ridge flank. *Front. Microbiol.* 7:820 10.3389/fmicb.2016.00820 PMC4882963 27303398

[B42] Jungbluth S. P. Bowers R. M. Lin H.-T. Cowen J. P. Rappé M. S. (2016). Novel microbial assemblages inhabiting crustal fluids within mid-ocean ridge flank subsurface basalt. *ISME J.* 10 2033–2047. 10.1038/ismej.2015.248 26872042 PMC5029167

[B43] Jungbluth S. P. Grote J. Lin H.-T. Cowen J. P. Rappe M. S. (2012). Microbial diversity within basement fluids of the sediment-buried Juan de Fuca Ridge flank. *ISME J.* 7 161–172. 10.1038/ismej.2012.73 22791235 PMC3526168

[B44] Jungbluth S. P. Johnson L. G. H. Cowen J. P. Rappé M. S. (2013). “Data report: microbial diversity in sediment nearby Grizzly Bare seamount from sites U1363B and U1363G,” in *Proceedings of the Integrated Ocean Drilling Program* 327 (College Station, TX: IODP-MI). 10.2204/iodp.proc.327.201.2013

[B45] Juretschko S. Timmermann G. Schmid M. Schleifer K.-H. Pommerening-Röser A. Koops H.-P. (1998). Combined molecular and conventional analyses of nitrifying bacterium diversity in activated sludge: *Nitrosococcus mobilis* and *Nitrospira*-like Bacteria as dominant populations. *Appl. Environ. Microbiol.* 64 3042–3051. 9687471 10.1128/aem.64.8.3042-3051.1998 PMC106813

[B46] Kallmeyer J. Pockalny R. Adhikari R. R. Smith D. C. D’Hondt S. (2012). Global distribution of microbial abundance and biomass in subseafloor sediment. *PNAS* 109 16213–16216. 10.1073/pnas.1203849109 22927371 PMC3479597

[B47] Kallmeyer J. Smith D. C. Spivack A. J. D’Hondt S. (2008). New cell extraction procedure applied to deep subsurface sediments. *Limnol. Oceanogr. Methods* 6 236–245. 10.4319/lom.2008.6.236

[B48] Kaster A.-K. Mayer-Blackwell K. Pasarelli B. Spormann A. M. (2014). Single cell genomic study of *Dehalococcoidetes* species from deep-sea sediments of the Peruvian Margin. *ISME J.* 8 1831–1842. 10.1038/ismej.2014.24 24599070 PMC4139717

[B49] LaRowe D. E. Amend J. P. (2014). “Energetic constraints on life in marine deep sediments,” in *Life in Extreme Environments: Microbial Life in the Deep Biosphere* eds Kallmeyer J. Wagner D. (Berlin: De Gruyter Publishing) 279–302.

[B50] Lee M. D. Walworth N. G. Sylvan J. B. Edwards K. J. Orcutt B. N. (2015). Microbial communities on seafloor basalts at Dorado Outcrop reflect level of alteration and highlight global lithic clades. *Front. Microbiol.* 6:1470 10.3389/fmicb.2015.01470 PMC4688349 26779122

[B51] Lever M. Alperin M. J. Engelen B. Inagaki F. Nakagawa S. Steinsbu B. O. (2006). Trends in basalt and sediment core contamination during IODP Expedition 301. *Geomicrobiol. J.* 23 517–530. 10.1080/01490450600897245

[B52] Lever M. A. (2013). Functional gene surveys from ocean drilling expeditions - a review and perspective. *FEMS Microbiol. Ecol.* 84 1–23. 10.1111/1574-6941.12051 23228016

[B53] Lever M. A. Alperin M. J. Teske A. Heuer V. Schmidt F. Hinrichs K. U. (2010). Acetogenesis in deep subseafloor sediments of the Juan du Fuca Ridge Flank: a synthesis of geochemical, thermodynamic, and gene-based evidence. *Geomicrobiol. J.* 27 183–211. 10.1080/01490450903456681

[B54] Lever M. A. Rouxel O. J. Alt J. C. Shimizu N. Ono S. Coggon R. M. (2013). Evidence for microbial carbon and sulfur cycling in deeply buried ridge flank basalt. *Science* 339 1305–1308. 10.1126/science.1229240 23493710

[B55] Lever M. A. Torti A. Eickenbusch P. Michaud A. B. Šantl-Temkiv T. Jørgensen B. B. (2015). A modular method for the extraction of DNA and RNA, and the separation of DNA pools from diverse environmental samples. *Front. Microbiol.* 6:476 10.3389/fmicb.2015.00476 PMC4436928 26042110

[B56] Li Y. Kawamura Y. Fujiwara N. Naka T. Liu H. Huang X. (2004). *Rothia aeria* sp. nov., *Rhodococcus baikonurensis* sp. nov., and *Arthrobacter russicus* sp. nov., isolated from air in the Russian space laboratory Mir. *Int. J. Syst. Evol. Microbiol.* 54 827–835. 10.1099/ijs.0.02828-0 15143031

[B57] Lin H.-T. Cowen J. P. Olson E. J. Amend J. P. Lilley M. D. (2012). Inorganic chemistry, gas compositions and dissolved organic carbon in fluids from sedimented young basaltic crust on the Juan de Fuca Ridge flanks. *Geochim. Cosmochim. Acta* 85 213–227. 10.1016/j.gca.2012.02.017

[B58] Lin H.-T. Hsieh C.-C. Cowen J. P. Rappé M. S. (2015). “Data report: dissolved and particulate organic carbon in the deep sediments of IODP Site U1363 near Grizzly Bare seamount,” in *Proceedings of the IODP* Vol. 327 eds Fisher A. T. Tsuji T. Petronotis K. Expedition 327 Scientists (Tokyo: Integrated Ocean Drilling Program Management International, Inc.).

[B59] Lysnes K. Thorseth I. H. Steinsbu B. O. Øvreås L. Torsvik T. Pedersen R. B. (2004). Microbial community diversity in seafloor basalt from the Arctic spreading ridges. *FEMS Microbiol. Ecol.* 50 213–230. 10.1016/j.femsec.2004.06.014 19712362

[B60] Marteinsson V. T. Rúnarsson A. Stefánsson A. Thorsteinsson T. Jóhannesson T. Magnússon S. (2012). Microbial communities in the subglacial waters of the Vatnajökull ice cap, Iceland. *ISME J.* 7 427–437. 10.1038/ismej.2012.97 22975882 PMC3554413

[B61] Mason O. U. Di Meo-Savoie C. A. Van Nostrand J. D. Zhou J. Fisk M. R. Giovannoni S. J. (2009). Prokaryotic diversity, distribution, and insights into their role in biogeochemical cycling in marine basalts. *ISME J.* 3 231–242. 10.1038/ismej.2008.92 18843298

[B62] Masui N. Morono Y. Inagaki F. (2008). Microbiological assessment of circulation mud fluids during the first operation of riser drilling by the Deep-Earth Research Vessel Chikyu. *Geomicrobiol. J.* 25 274–282. 10.1080/01490450802258154

[B63] Meyer J. L. Jaekel U. Tully B. J. Glazer B. T. Wheat C. G. Lin H.-T. (2016). A distinct and active bacterial community in cold oxygenated fluids circulating beneath the western flank of the Mid-Atlantic ridge. *Sci. Rep.* 6:22541 10.1038/srep22541 PMC4776111 26935537

[B64] Morono Y. Terada T. Nishizawa M. Ito M. Hillion F. Takahata N. (2011). Carbon and nitrogen assimilation in deep subseafloor microbial cells. *Proc. Natl. Acad. Sci. U.S.A.* 108 18295–18300. 10.1073/pnas.1107763108 21987801 PMC3215001

[B65] Orcutt B. N. Bach W. Becker K. Fisher A. T. Hentscher M. Toner B. M. (2011a). Colonization of subsurface microbial observatories deployed in young ocean crust. *ISME J.* 5 692–703. 10.1038/ismej.2010.157 21107442 PMC3217339

[B66] Orcutt B. N. Sylvan J. B. Knab N. J. Edwards K. J. (2011b). Microbial ecology of the dark ocean above, at, and below the seafloor. *Microbiol. Mol. Biol. Rev.* 75 361–422. 10.1128/MMBR.00039-10 21646433 PMC3122624

[B67] Orcutt B. N. Wheat C. G. Rouxel O. J. Hulme S. Edwards K. J. Bach W. (2013). Oxygen consumption rates in subseafloor basaltic crust derived from a reaction transport model. *Nat. Commun.* 4:2539 10.1038/ncomms3539 24071791

[B68] Orsi W. D. Edgcomb V. P. Christman G. D. Biddle J. F. (2013). Gene expression in the deep biosphere. *Nature* 499 205–208. 10.1038/nature12230 23760485

[B69] Pruesse E. Peplies J. Glockner F. O. (2012). SINA: accurate high-throughput multiple sequence alignment of ribosomal RNA genes. *Bioinformatics* 28 1823–1829. 10.1093/bioinformatics/bts252 22556368 PMC3389763

[B70] Rappé M. S. Connon S. A. Vergin K. L. Giovannoni S. J. (2002). Cultivation of the ubiquitous SAR11 marine bacterioplankton clade. *Nature* 418 630–633. 10.1038/nature00917 12167859

[B71] Rinke C. Schwientek P. Sczyrba A. Ivanova N. N. Anderson I. J. Cheng J.-F. (2013). Insights into the phylogeny and coding potential of microbial dark matter. *Nature* 499 431–437. 10.1038/nature12352 23851394

[B72] Røy H. Kallmeyer J. Adhikar R. R. Pockalny R. Jørgensen B. B. D’Hondt S. (2012). Aerobic microbial respiration in 86-million-year-old deep-sea red clay. *Science* 336 922–925. 10.1126/science.1219424 22605778

[B73] Salter S. J. Cox M. Turek E. M. Calus S. T. Cookson W. O. Moffatt M. F. (2014). Reagent and laboratory contamination can critically impact sequence-based microbiome analyses. *BMC Biol.* 12:87 10.1186/s12915-014-0087-z PMC4228153 25387460

[B74] Santelli C. M. Banerjee N. Bach W. Edwards K. J. (2010). Tapping the subsurface ocean crust biosphere: low biomass and drilling-related contamination calls for improved quality controls. *Geomicrobiol. J.* 27 158–169. 10.1080/01490450903456780

[B75] Santelli C. M. Orcutt N. Banning E. Bach W. Moyer C. L. Sogin M. L. (2008). Abundance and diversity of microbial life in ocean crust. *Nature* 453 653–656. 10.1038/nature06899 18509444

[B76] Schloss P. D. Gevers D. Westcott S. L. (2011). Reducing the effects of PCR amplification and sequencing artifacts on 16S rRNA-based studies. *PLoS ONE* 6:e27310 10.1371/journal.pone.0027310 PMC3237409 22194782

[B77] Schloss P. D. Westcott S. L. Ryabin T. Hall J. R. Hartmann M. Hollister E. B. (2009). Introducing *mothur*: open-source, platform-independent, community-supported software for describing and comparing microbial communities. *Appl. Environ. Microbiol.* 75 7537–7541. 10.1128/AEM.01541-09 19801464 PMC2786419

[B78] Smith A. Popa R. Fisk M. R. Nielsen M. Wheat C. G. Jannasch H. W. (2011). In situ enrichment of ocean crust microbes on igneous minerals and glasses using an osmotic flow-through device. *Geochem. Geophys. Geosyst.* 12:Q06007 10.1029/2010gc003424

[B79] Smith A. R. Fisk M. R. Thurber A. R. Flores G. Mason O. U. Popa R. (2016). Deep crustal communities of the Juan de Fuca Ridge are governed by mineralogy. *Geomicrobiol. J.* 34 147–156. 10.1080/01490451.2016.1155001

[B80] Smith D. C. Spivack A. J. Fisk M. R. Haveman S. A. Staudigel H. Party L. S. S. (2000). Methods for quantifying potential microbial contamination during deep ocean coring. *ODP Tech. Note* 28:19 10.2973/odp.tn.28.2000

[B81] Sogin M. L. Morrison H. G. Huber J. A. Welch D. M. Huse S. M. Neal P. R. (2006). Microbial diversity in the deep sea and the underexplored “rare biosphere”. *Proc. Natl. Acad. Sci. U.S.A.* 103 12115–12120. 10.1073/pnas.0605127103 16880384 PMC1524930

[B82] Stoddard S. F. Smith B. J. Hein R. Roller B. R. K. Schmidt T. M. (2015). *rrnDB*: improved tools for interpreting rRNA gene abundance in bacteria and archaea and a new foundation for future development. *Nucleic Acids Res.* 43 D593–D598. 10.1093/nar/gku1201 25414355 PMC4383981

[B83] Süß J. Schubert K. Sass H. Cypionka H. Overmann J. Engelen B. (2006). Widespread distribution and high abundance of *Rhizobium radiobacter* within Mediterranean subsurface sediments. *Environ. Microbiol.* 8 1753–1763. 10.1111/j.1462-2920.2006.01058.x 16958756

[B84] Thorseth I. H. Pedersen R. B. Christie D. M. (2003). Microbial alteration of 0-30-Ma seafloor and sub-seafloor basaltic glasses from the Australian Antarctic Discordance. *Earth Planet. Sci. Lett.* 215 237–247. 10.1016/S0012-821X(03)00427-8

[B85] Wankel S. D. Buchwald C. Ziebis W. Wenk C. B. Lehmann M. F. (2015). Nitrogen cycling in the deep sedimentary biosphere: nitrate isotopes in porewaters underlying the oligotrophic North Atlantic. *Biogeosciences* 12 7483–7502. 10.5194/bg-12-7483-2015

[B86] Wasmund K. Cooper M. Schreiber L. Lloyd K. G. Baker B. J. Petersen D. G. (2016). Single-cell genome and group-specific *dsrAB* sequencing implicate marine members of the Class *Dehalococcoidia* (Phylum Chloroflexi) in sulfur cycling. *mBio* 7:e00266-16 10.1128/mBio.00266-16 PMC4959651 27143384

[B87] Wasmund K. Schreiber L. Lloyd K. G. Petersen D. G. Schramm A. Stepanauskas R. (2014). Genome sequencing of a single cell of the widely distributed marine subsurface *Dehalococcoidia*, phylum *Chloroflexi*. *ISME J.* 8 383–397. 10.1038/ismej.2013.143 23966099 PMC3906807

[B88] Wheat C. G. Hulme S. M. Fisher A. T. Orcutt B. N. Becker K. (2013). Seawater recharge into oceanic crust: IODP Exp 327 Site U1363 Grizzly Bare Outcrop. *Geochem. Geophys. Geosyst.* 14 1957–1972. 10.1002/ggge.20131

[B89] Wheat C. G. Mottl M. J. (2000). Composition of pore and spring waters from Baby Bare: global implications of geochemical fluxes from a ridge flank hydrothermal system. *Geochim. Cosmochim. Acta* 64 629–642. 10.1016/S0016-7037(99)00347-6

[B90] Wheat C. G. Mottl M. J. (2004). “Geochemical fluxes through mid-ocean ridge flanks,” in *Hydrogeology of the Oceanic Lithosphere* eds Davis E. E. Elderfield H. (Cambridge: Cambridge University Press) 627–658.

[B91] Winslow D. M. Fisher A. T. (2015). Sustainability and dynamics of outcrop-to-outcrop hydrothermal circulation. *Nat. Commun.* 6:7567 10.1038/ncomms8567 PMC4491839 26113260

[B92] Yilmaz P. Parfrey L. W. Yarza P. Gerken J. Pruesse E. Quast C. (2014). The SILVA and “all-species Living Tree Project (LTP)” taxonomic frameworks. *Nucleic Acids Res.* 42 643–648. 10.1093/nar/gkt1209 24293649 PMC3965112

[B93] Yu Y. Lee C. Kim J. Hwang S. (2005). Group-specific primer and probe sets to detect methanogenic communities using quantitative real-time polymerase chain reaction. *Biotechnol. Bioeng.* 89 670–679. 10.1002/bit.20347 15696537

[B94] Zhang X. Feng X. Wang F. (2016). Diversity and metabolic potential of subsurface crustal microorganisms from the western flank of the Mid-Atlantic Ridge. *Front. Microbiol.* 7:363 10.3389/fmicb.2016.00363 PMC4797314 27047476

[B95] Ziebis W. McManus J. Ferdelman T. Schmidt-Schierhorn F. Bach W. Muratli J. (2012). Interstitial fluid chemistry of sdiments underlying the North Atlantic Gyre and the influence of subsurface fluid flow. *Earth Planet. Sci. Lett.* 32 79–91. 10.1016/j.epsl.2012.01.018

