# Supplementary figures and images for: Influence of Igneous Basement on Deep Sediment Microbial Diversity on the Eastern Juan de Fuca Ridge Flank
Source: Front Microbiol. 2017 Aug 2;8:1434. doi: 10.3389/fmicb.2017.01434 (PMC5539551; doi:10.3389/fmicb.2017.01434)

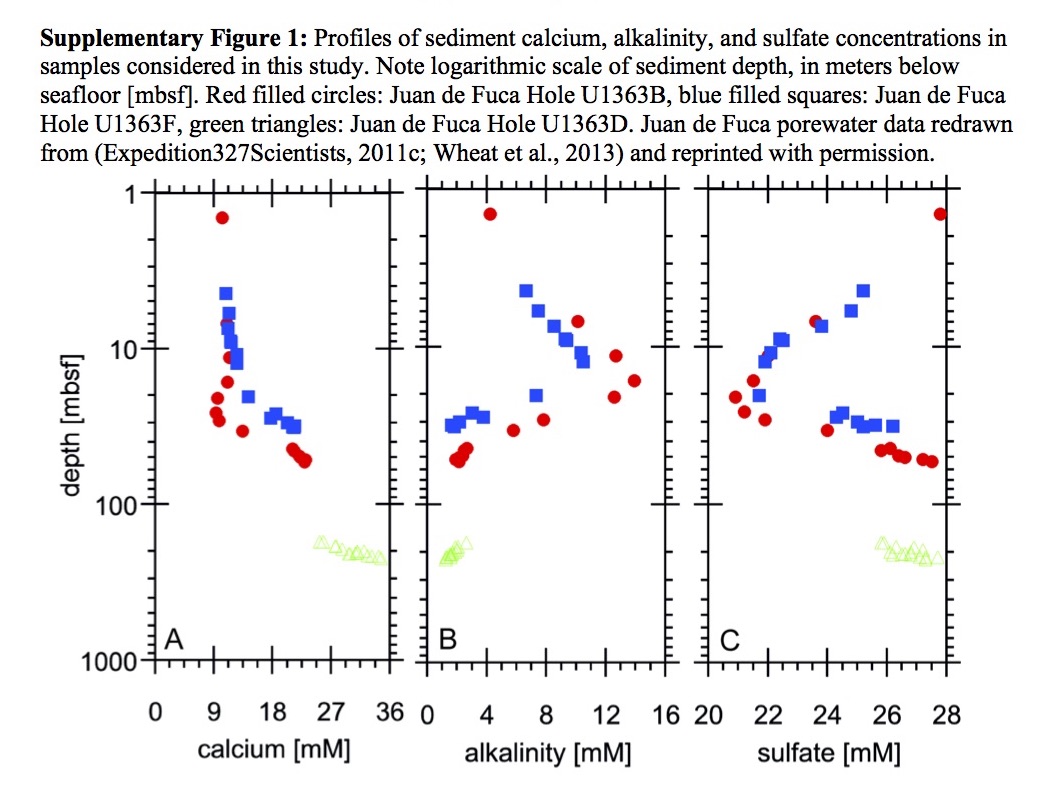

Supplement: Supplementary file 2 [file Image_1.JPEG]

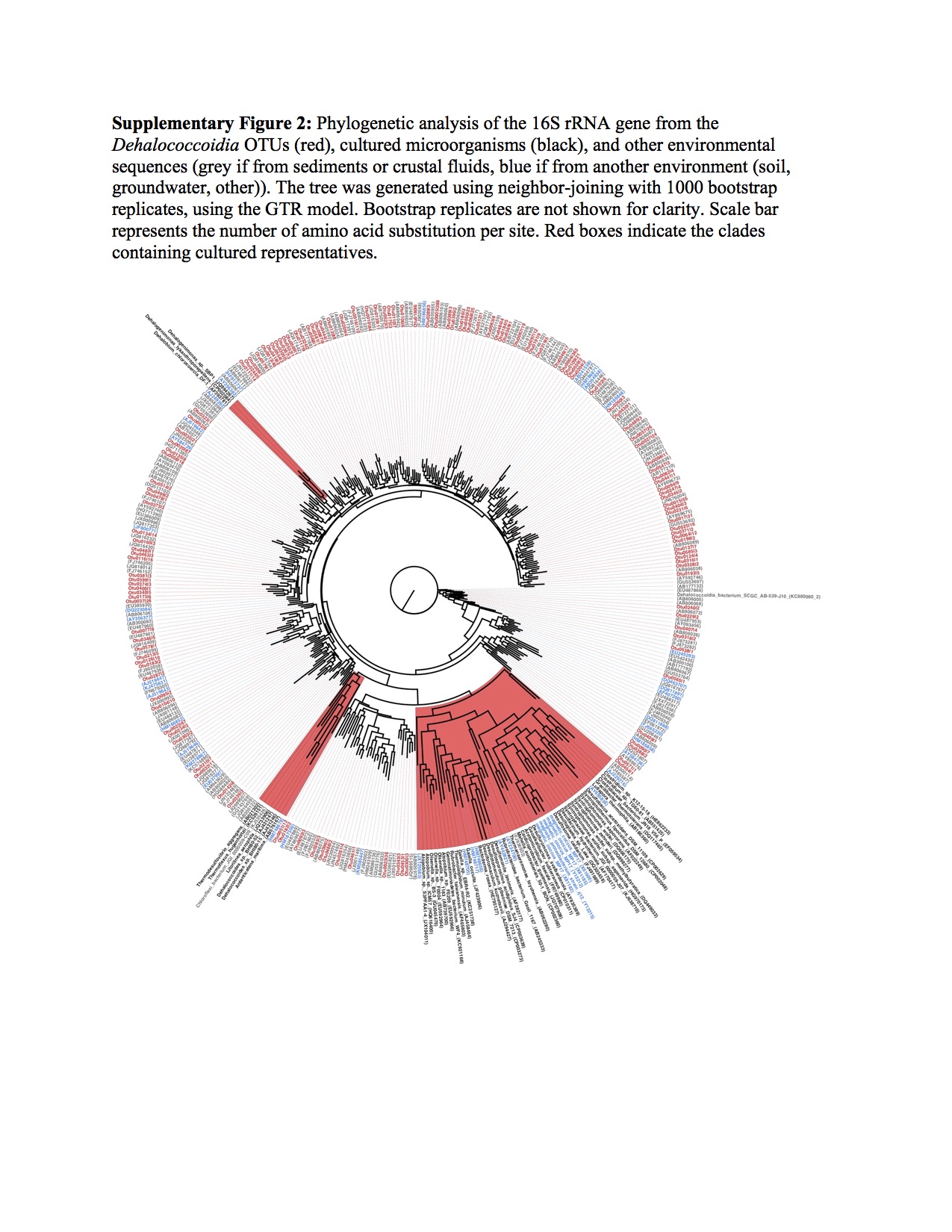

Supplement: Supplementary file 3 [file Image_2.JPEG]

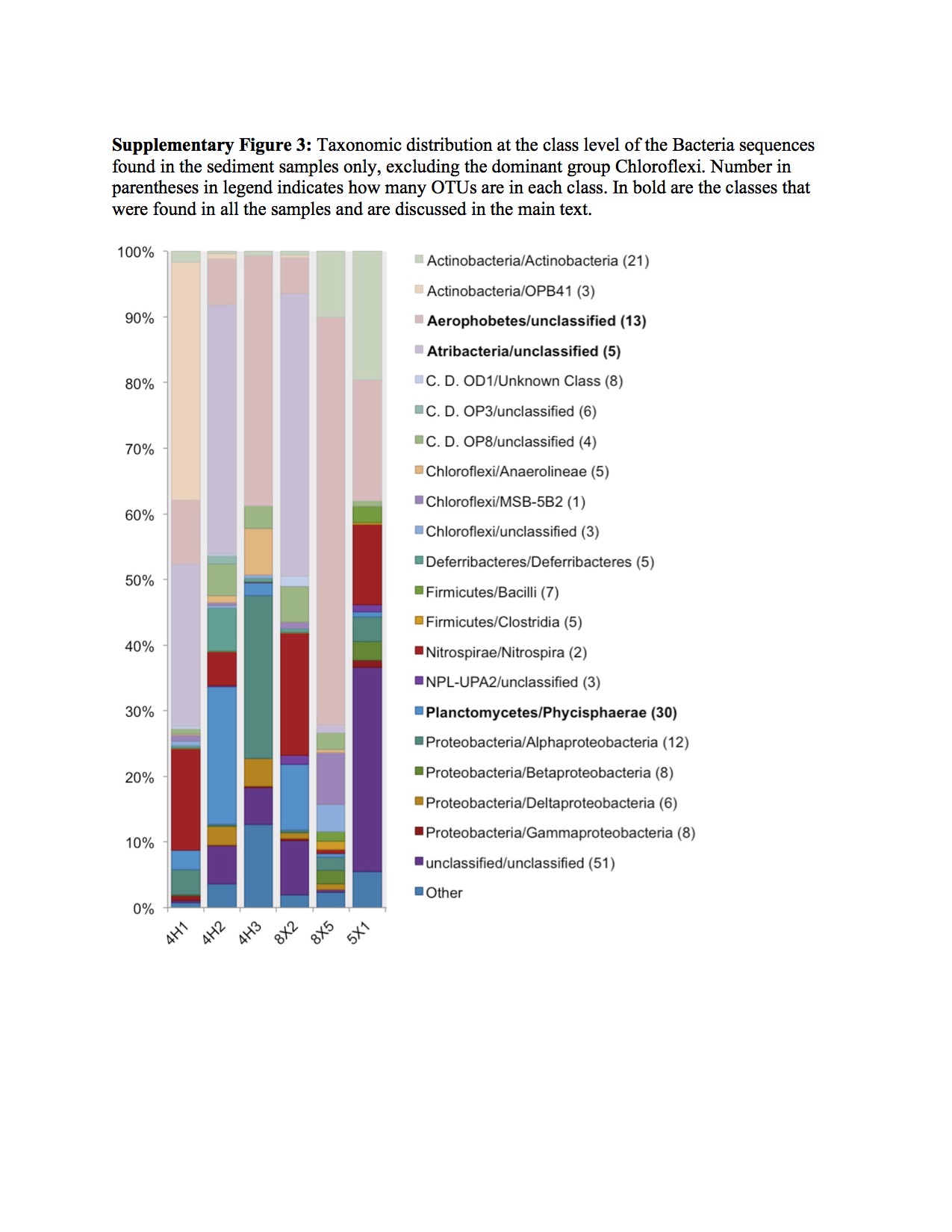

Supplement: Supplementary file 4 [file Image_3.JPEG]
